# Stability study in selected conditions and biofilm-reducing activity of phages active against drug-resistant *Acinetobacter baumannii*

**DOI:** 10.1038/s41598-024-54469-z

**Published:** 2024-02-21

**Authors:** Natalia Bagińska, Ilona Grygiel, Filip Orwat, Marek Adam Harhala, Adam Jędrusiak, Elżbieta Gębarowska, Sławomir Letkiewicz, Andrzej Górski, Ewa Jończyk-Matysiak

**Affiliations:** 1 Bacteriophage Laboratory, Hirszfeld Institute of Immunology and Experimental Therapy, Polish Academy of Sciences, 53-114 Wrocław, Poland; 2 Laboratory of Phage Molecular Biology, Hirszfeld Institute of Immunology and Experimental Therapy, Polish Academy of Sciences, 53-114 Wrocław, Poland; 3 Division of Biogeochemistry and Environmental Microbiology, Department of Plant Protection, Wroclaw University of Environmental and Life Sciences, Grunwaldzka 53, 50-357 Wrocław, Poland; 4 Phage Therapy Unit, Hirszfeld Institute of Immunology and Experimental Therapy, Polish Academy of Sciences, 53-114 Wrocław, Poland; 5 Collegium Medicum, Jan Długosz University, Częstochowa, Poland

**Keywords:** Microbiology, Diseases, Risk factors

## Abstract

*Acinetobacter baumannii* is currently a serious threat to human health, especially to people with immunodeficiency as well as patients with prolonged hospital stays and those undergoing invasive medical procedures. The ever-increasing percentage of strains characterized by multidrug resistance to widely used antibiotics and their ability to form biofilms make it difficult to fight infections with traditional antibiotic therapy. In view of the above, phage therapy seems to be extremely attractive. Therefore, phages with good storage stability are recommended for therapeutic purposes. In this work, we present the results of studies on the stability of 12 phages specific for *A. baumannii* under different conditions (including temperature, different pH values, commercially available disinfectants, essential oils, and surfactants) and in the urine of patients with urinary tract infections (UTIs). Based on our long-term stability studies, the most optimal storage method for the A. baumannii phage turned out to be − 70 °C. In contrast, 60 °C caused a significant decrease in phage activity after 1 h of incubation. The tested phages were the most stable at a pH from 7.0 to 9.0, with the most inactivating pH being strongly acidic. Interestingly, ethanol-based disinfectants caused a significant decrease in phage titers even after 30 s of incubation. Moreover, copper and silver nanoparticle solutions also caused a decrease in phage titers (which was statistically significant, except for the Acba_3 phage incubated in silver solution), but to a much lesser extent than disinfectants. However, bacteriophages incubated for 24 h in essential oils (cinnamon and eucalyptus) can be considered stable.

## Introduction

An opportunistic Gram-negative bacterial species, *Acinetobacter baumannii*, belongs to the ESKAPE group (containing: *Enterococcus faecium*, *Staphylococcus aureus*, *Klebsiella pneumoniae*, *A. baumannii*, *Pseudomonas aeruginosa*, *Enterobacter* spp.), which includes the most dangerous pathogenic bacteria because of their resistance to antibiotics^1^. Research shows that the use of antibiotics in Wroclaw hospitals in the years 2020–2022 increased significantly, which translated to an increase in the resistance of *A. baumannii* strains^2^, among others. Those strains that are widely resistant to commonly used antibiotics particularly pose a threat both to human health and life worldwide. Therefore, *A. baumannii* was covered in World Health Organization (WHO) reports, where these bacteria were classified as a critical threat priority among multi-drug resistant (MDR) bacteria (which may be resistant to third generation cephalosporins and carbapenems) and necessitates the search for new agents effective in the fight against these bacteria^3,4^. *A. baumannii* can cause life-threatening infections, in particular, in hospital wards. These infections can affect the soft tissues, urinary tract, lung, skin or bloodstream. People in critical condition in intensive care units (ICUs) and those with immunodeficiency are particularly at risk^5–9^. The percentage of infections caused by *A. baumannii* strains in ICUs worldwide is as high as 20%^10,11^. The reason for this phenomenon is the resistance of the strains to external/environmental factors as well as broad resistance to antibiotics^12^. It is worth mentioning that the mortality rate among people infected with these strains is higher than for infections with other gram-negative bacteria (e.g., *K. pneumoniae*)^13,14^. Genomic and phenotypic analysis of *A. baumannii* strains identified that the pathogenicity and toxicity among these strains has increased^1,6^. The virulence factors that are responsible for this phenomenon have also been described as: capsular polysaccharides, iron-chelating systems, lipopolysaccharides, outer membrane porins, phospholipases, proteases and protein secretion systems^5,15–18^. Additionally, bacteria use various mechanisms to defend themselves against phages. Hyperactivation of the global regulator BfmRS in a Loki phage sensitive to *A. baumannii* 17,978 has been studied to increase phage adsorption to bacterial cells, phage replication and thus bacterial killing. In turn, inactivation of the BfmRS inhibits Loki phage adsorption and killing of *A. baumannii*. Interestingly, mutations of the bacterial glycosyltransferase responsible for capsule structure and bacterial virulence can lead to complete resistance of *A. baumannii* to the Loki phage^19^.

An important factor determining the strong expansion of *A. baumannii* strains in hospital environments is their ability to acquire resistance to currently used antibiotics. This is the result of genetic mutations, horizontal acquisition of resistance genes, as well as their amplification and expression^12^. The emergence of carbapenem-resistant *A. baumannii* (CRAB) is particularly dangerous, as it necessitates the use of the last resort antibiotics tigecycline or colistin^20^. However, resistance to these antibiotics has also been noted^21–23^. It is worth mentioning that *A. baumannii* has the ability to form a biofilm on various surfaces, including host tissues and surfaces of hospital equipment (e.g., urinary catheters), which further hinders the control of this pathogen^24–26^. Interestingly, bacteria forming a biofilm can coexist with other microorganisms (e.g., fungi), which additionally complicates the fight against these pathogens, protecting them against the action of antibiotics, disinfectants and immune system cells^27^. Conducted research shows that the use of bacteriophages/their enzymes allows for better penetration of the biofilm matrix and combating multimicrobial biofims.

In recent years, there have been new studies on phages specific for *A. baumannii*, especially those strains that are resistant to commonly used antibiotics. By October 2022, 132 *A. baumannii*-specific phages had been tested and the studies showed that their genomes ranged from 4 to 234 kb^28^. Wintachai et al. isolated and characterized the lytic phage vB_AbaM_ABPW7 (vABPW7) MDR-specific for *A. baumannii* ABPW063, which is characterized by rapid adsorption (after 15 min of incubation, more than 95% of the phage particles adsorbed to the host cell)^29^. The phage was stable under various conditions (temperature, pH, presence of glycerol, UV) had a broad lytic spectrum and in vitro studies have shown that it is also effective in reducing bacterial biofilm. However, studies in the human A549 alveolar epithelial cell culture model have shown that the phage contributes to reduced adhesion of *A. baumannii* MDR bacterial cells. Additionally, a cytotoxic effect of the phage has not been observed for the tested cells. Another lytic phage specific for *A. baumannii* with therapeutic potential is the Abp95 phage isolated from hospital sewage that is characterized by a relatively broad lytic spectrum (of 29%) and its genome size was estimated at 43.176 bp^30^. Additionally, no lysogeny, virulence or antibiotic resistance genes were found in its genome. Studies on a diabetic mouse model of wound infection have shown that the use of phage solution in phosphate-buffered saline (PBS), when applied directly to the wound, promotes the removal of infections caused by MDR strains of *A. baumannii*, and thus accelerates wound healing.

An interesting way of applying phages may be their use as a biological control agent against CRAB. Chen et al*.* described the use of a phage cocktail in an aerosol in ICUs in the medical center in Taiwan^31^. Prior to the use of the cocktails to decontaminate the space used by a patient diagnosed with infection caused by CRAB strains, phage typing of 501 *A. baumannii* strains was carried out to develop the optimal composition of phage cocktails. Interestingly, the percentage of CRAB strains present in intensive care units decreased significantly from 65.3 to 55% within 3 years of decontamination (2017–2019). The cited studies suggest the potential of using phages also as a disinfecting agent in the fight with *A. baumannii* strains. An interesting and effective solution may also be the application of bacteriophages as gel hand disinfectant containing the ɸPT1b phage (active against *Escherichia coli*) with hydroxypropyl methylcellulose (HPMC) and active glycerin ingredients^32^.

In addition to the whole phages themselves, enzymes that are encoded in their genomes also have antibacterial potential. Such an example is the AbEndolysin with an *N*-acetylmuramidase-containing catalytic domain which was identified in the genome of bacteriophage ΦAb1656-2 induced with the use of mitomycin C from the *A. baumannii* 1656-2 strain (isolated from recovered hospitalized patients). The obtained prophage was capable of infecting MDR strains of *A. baumannii*. Also recombinant, purified endolysin showed antimicrobial activity against MDR *A. baumannii* strains, which makes it a promising candidate for phage therapy of infections caused by *A. buamannii* strains^33^.

In the current work we aimed to evaluate the therapeutic potential of *A. baumannii* specific phages based on their ability to retain activity under different conditions or incubation with the presence of factors with an antibacterial potential.

## Results

### Bacterial strains and bacteriophages

During the research, 257 strains belonging to *Acinetobacter* spp. were used (*Acinetobacter ursingii* n = 1, *Acinetobacter pittii* n = 2, *Acinetobacter johnsonii* n = 2, and *A. baumannii* n = 252), most of which were resistant to commonly used antibiotics: ciprofloxacin, levofloxacin, trimethoprim/sulfamethoxazole, gentamicin, netilmicin, tobramycin, tigecycline, colistin, imipenem, and meropenem. Ten of them (Table 1) were used for biofilm research, the rest were used to search for bacteriophages specific to *A. baumannii* and to determine the biological properties of isolated phages^34^. The strains were isolated from four main sources: respiratory 41%, urinary tract 17%, soft tissue/skin 15% and blood 10%. In a previous work, 12 isolated *A. baumannii*-specific phages were first genetically and biologically characterized. The characterized phages were isolated from liquid samples, including city ditch water, hospital sewage, sewage treated at a sewage treatment plant, river water, and water from a drainage ditch. Interestingly, the only lytic phage (Acba_6) was isolated from a hospital wastewater sample, which has been suggested to be a good source of *A. baumannii*-specific phage isolation^35^. The lytic spectrum of the tested phages ranged from 11 (Acba_1, Aclw_8, Acba_18) to 75% (Acba_15)^34^. On the basis of genetic studies, the Acba_6 phage was classified as a lytic phage (belonging to *Junivirinae* subfamily), while the remaining phage was classified as a temperate phage (belonging to *Beijernickvirinae* subfamily). Additionally, the size of the genomes of the characterized phages ranged from 32,415 bp—Aclw_9 to 67,052 bp—Acba_11.

**Table 1:** Source of isolation and resistance of *Acinetobacter baumannii* strains used for biofilm research.

No.	Strain name	Source	CIP	LVX	SXT	AN	GN	NET	TOB	TGC	CS	IMI	MRP
1	Ab1	Urine	R	R	R	R	R	R	S	R	S	R	R
2	Ab2	Urine	R	R	R	R	R	R	S	R	S	R	R
3	Ab3	Urine	R	R	R	R	R	R	R	R	S	R	R
4	Ab10	Urine	R	R	R	R	R	R	R	R	S	R	S
5	Ab12	Urine	R	S	S	S	R	R	R	R	S	R	R
6	Ab13	Urine	R	R	R	R	R	R	R	R	S	R	R
7	Ab39	Urine	R	R	R	R	R	R	R	R	S	R	R
8	Ab57	Catheter	R	R	R	R	R	S	R	R	S	R	R
9	Ab68	Urine	R	R	R	R	R	R	R	R	S	R	R
10	Ab82	Urine	R	R	R	R	R	R	R	R	S	R	R

*CIP* ciprofloxacin, *LVX* levofloxacin, *SXT* trimethoprim/sulfamethoxazole, *AN* amikacin, *GN* gentamycin, *NET* netilmicin, *TOB* tobramycin, *TGC* tigecycline, *CS* colistin, *IMI* imipenem, *MRP* meropenem, *R* resistant, *S* sensitive.

### Stability of isolated phages at various temperatures

**Figure 1:**
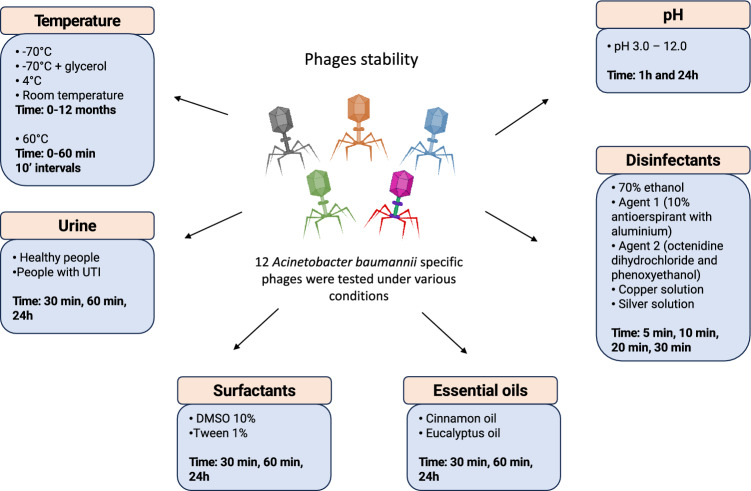
Stability studies of isolated phages specific to *A. baumannii* presented in this paper.

**Figure 2:**
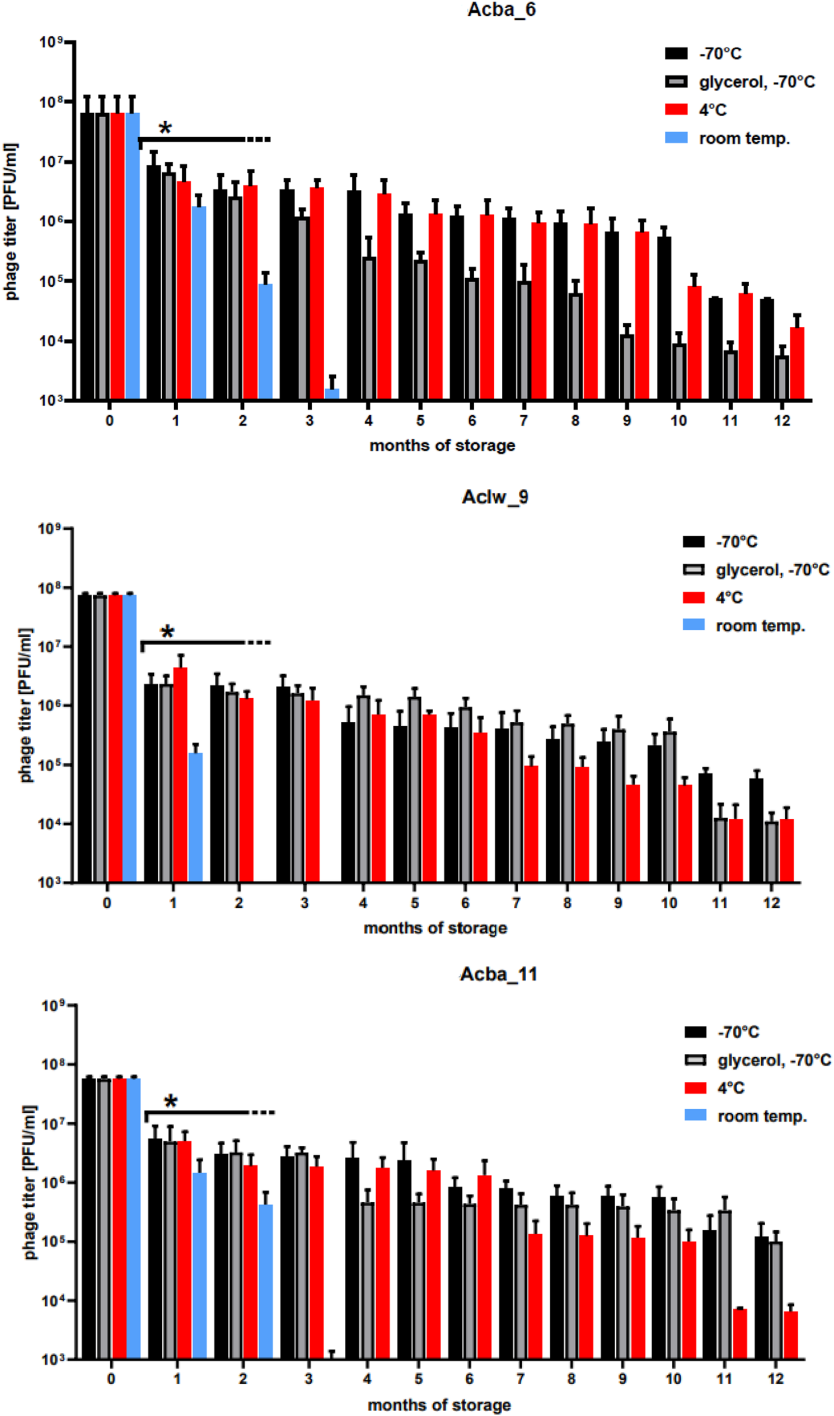
Stability of selected phages under different temperature conditions during a 12-month incubation period. Error bars represent the standard deviation (± SD) of the mean phage titers. *p < 0.05.

The results of phage stability tests carried out under various experimental conditions and presented in this paper are summarized in Fig. 1. Previously isolated phages (n = 12) (described in a paper by Bagińska et al.) were incubated for 12 months at 4 °C, room temperature (25 °C), − 70 °C and − 70 °C with the addition of 25% glycerol^34^. The decrease in the selected bacteriophage titer depending on the length of incubation at a given temperature is shown in Fig. 2. Room temperature turned out to be the least optimal for storage in the case of all tested phages—after 2 months of incubation the Aclw_9 phage titer decreased to 1.75 × 10^1^ PFU/mL (the initial titer 7.4 × 10^7^ PFU/mL), and after 3 months of incubation no active phage particles were detected. However, for Acba_6 and Acba_11, no active phages were detected after 4 months of incubation at room temperature. Moreover, after 12 months of incubation of the Acba_6 phage, its titer decreased by 4.1 orders of magnitude at 4 °C, 3.8 orders of magnitude at − 70 °C and 4.7 orders of magnitude at − 70 °C with 25% glycerol. For the Aclw_9 phage, a 12-month incubation period at 4 °C and − 70 °C with the addition of 25% glycerol resulted in a decrease in the titer relative to the baseline by 3.6 orders of magnitude, while − 70 °C resulted in a 3.2 order of magnitude reduction. Incubation of the Acba_11 phage at − 70 °C and − 70 °C with the addition of 25% glycerol resulted in a decrease in the titer relative to the baseline by about 2.4 orders of magnitude, and at 4 °C by about 3.9 orders. Results for the remaining 9 bacteriophages are included in the supplementary materials (Fig. S1). Titer decreases over 12 months of incubation from the baseline are statistically significant.

**Figure 3:**
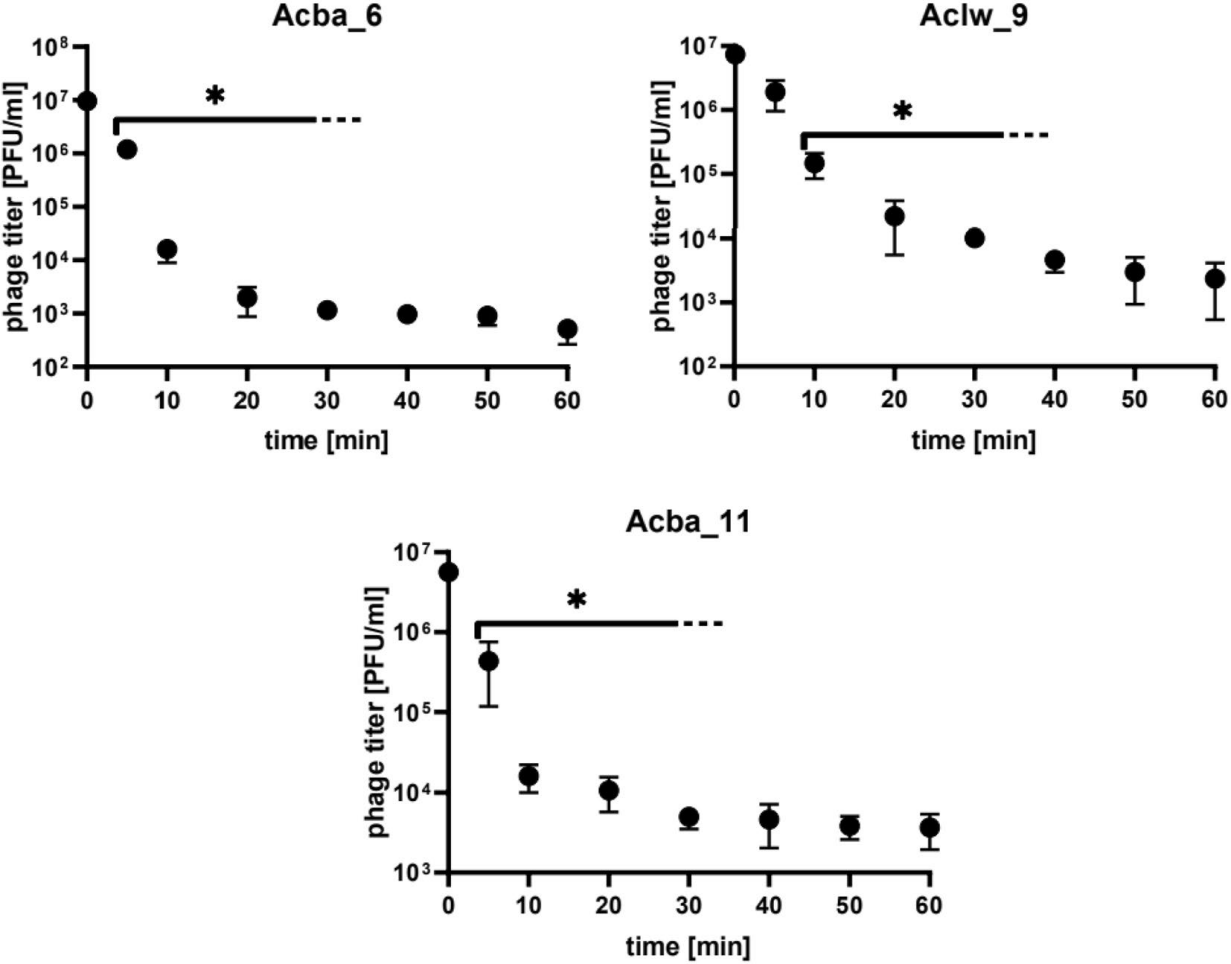
Stability of selected phages: Acba_6, Aclw_9, Acba_11 after one hour of incubation at 60 °C. Error bars represent the standard deviation (± SD) of the mean phage titers. *p < 0.05.

In addition, the stability of each phage at 60 °C for 1 h was tested (Fig. 3). Phage titers of Acba_6, Aclw_9 and Acba_11 decreased after one hour of incubation by approximately 4.4, 3.5 and 3.2 orders of magnitude, respectively. Titer decreases are statistically significant. Results for the remaining 9 bacteriophages are included in the supplement (Fig. S2).

### Stability of isolated phages at various pH values

**Figure 4:**
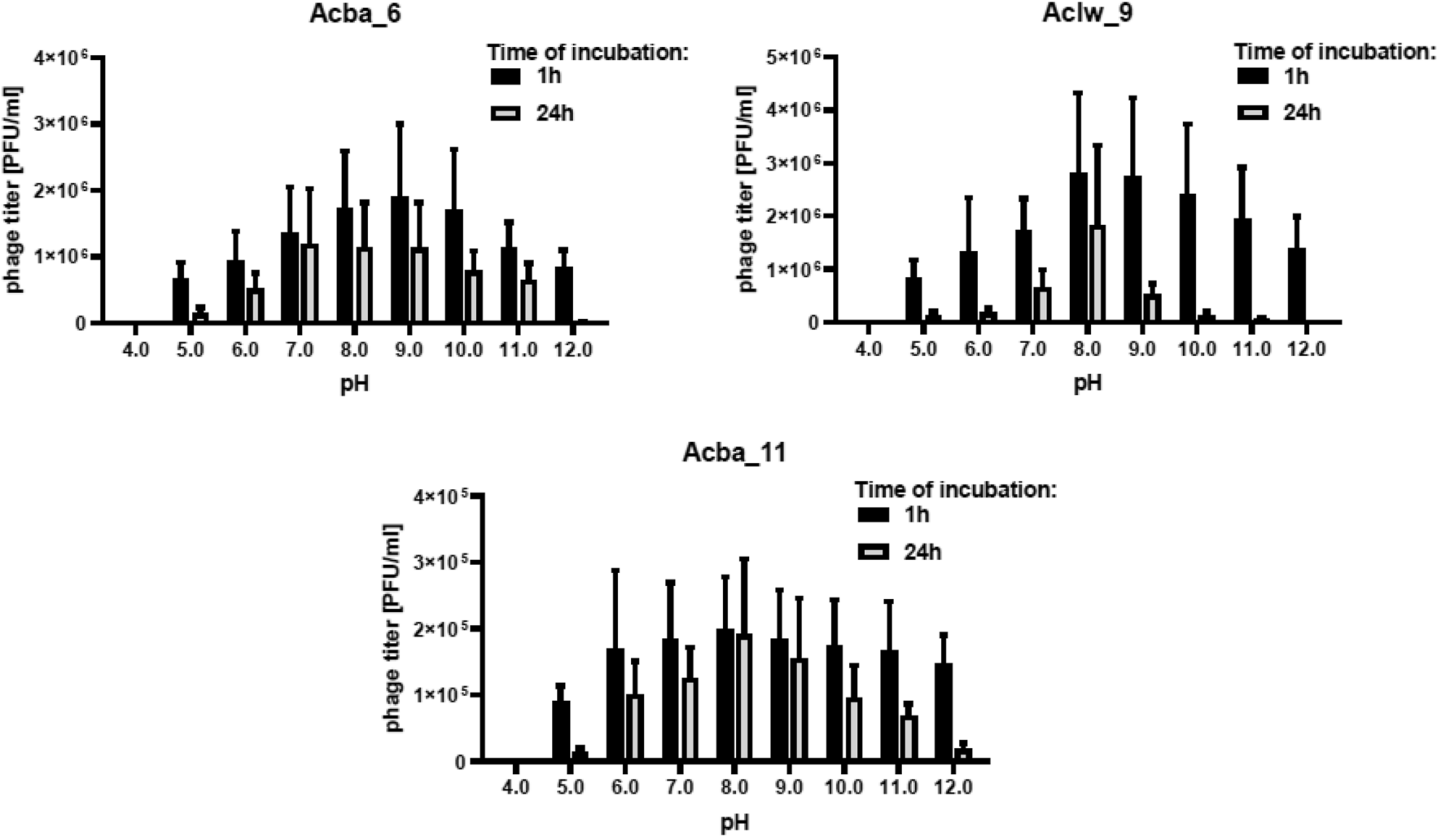
Stability of selected phages in various pH conditions during 1 h and 24 h of incubation. Error bars represent the standard deviation (± SD) of the mean phage titers.

In addition to studying the stability of phages at different temperatures, their ability to retain titer at different pH values was also tested (Fig. 4). Phage lysates were incubated for 1 h and 24 h in a solution with a given pH value. A solution with a pH of 7.0 was used as a reference point. The obtained results show that low pH solutions (pH 3.0–4.0) inactivate phages more strongly than alkaline (pH 11.0–12.0). Additionally, 24 h of incubation resulted in a greater decrease in phage titer than 1 h of incubation. The most optimal pH for the Acba_6 phage turned out to be pH 9.0 (both after 1 h and 24 h of incubation). However, for pH 3.0 and 4.0, no active phage particle was detected, regardless of the incubation time. On the other hand, for the Aclw_9 and Acba_11 phages, incubation in a solution with a pH of 8.0 caused the smallest decrease in the titer of these phages, regardless of the incubation time. For both of these phages, after 1 h of incubation, active phage particles were not detected at pH 3.0, while after 24 h of incubation, no active phage particles were detected in the solution at pH 4.0. The titer decreases are not statistically significant. Results for the remaining 9 bacteriophages are included in the supplement (Fig. S3).

### Phage stability in selected disinfectants and metal ions

Phage activity in selected agents commonly used for disinfection: 10% antiperspirant containing aluminum—agent 1, agent containing octenidine dihydrochloride and phenoxyethanol—agent 2, 70% ethanol, ethanol-based agents: agent 3 as well as agent 4 and solutions containing copper and silver nanoparticles were tested. The highest decrease in phage activity was observed for disinfectants containing ethanol and antiperspirants containing aluminum. Interestingly, agent 2 caused the fastest loss of the activity of selected phages such that after 5 min of incubation, no active phage particles were detected (results are not shown for the other phages). On the other hand, the phages better retained their activity when they were incubated in solutions of copper or silver nanoparticles (Table 2). The percentage decrease in the titer of selected phages is also shown in the supplement in the Figs. S4 and S5.

**Table 2:** Stability of selected phages in the presence of commonly used disinfectants and metal ions.

Phage symbol	Acba_3				Acba_4				Aclw_8			
Percentage of active phages after incubation with the selected agent (%)												
Tested factor/incubation time (min)	5	10	20	30	5	10	20	30	5	10	20	30
70% ethanol	0.37*	0.23*	0.13*	0.08*	0.24*	0.13*	0.12*	0.07*	0.20*	0.13*	0.04*	0.03*
Agent 1	0.05*	0.07*	0.04*	0.13*	0.08*	0.08*	0.09*	0.10*	n.a.p.*	n.a.p.*	n.a.p.*	n.a.p.*
Agent 2	n.a.p.*	n.a.p.*	n.a.p.*	n.a.p.*	n.a.p.*	n.a.p.*	n.a.p.*	n.a.p.*	n.a.p.*	n.a.p.*	n.a.p.*	n.a.p.*
Copper solution	67.91*	68.50	62.30	70.28*	42.12*	36.26*	39.21*	40.33*	58.68*	51.89*	73.95*	55.48*
Silver solution	99.80	86.22*	99.51*	98.62*	31.59*	32.05*	34.77*	39.40*	61.08*	49.10*	47.90*	49.10*
Percentage of active phages after incubation with the selected agent (%)												
Incubation time (s)	30		60		30		60		30		60	
Agent 3	3.24*		2.17*		2.21*		1.18*		3.70*		3.18*	
Agent 4	1.66*		1.20*		0.59*		0.35*		1.05*		0.82*	

*n.a.p.* no active phage particle was detected.

*Statistically significant decrease compared to the baseline titer, p < 0.05.

### Stability of phages in essential oils and surfactants

**Figure 5:**
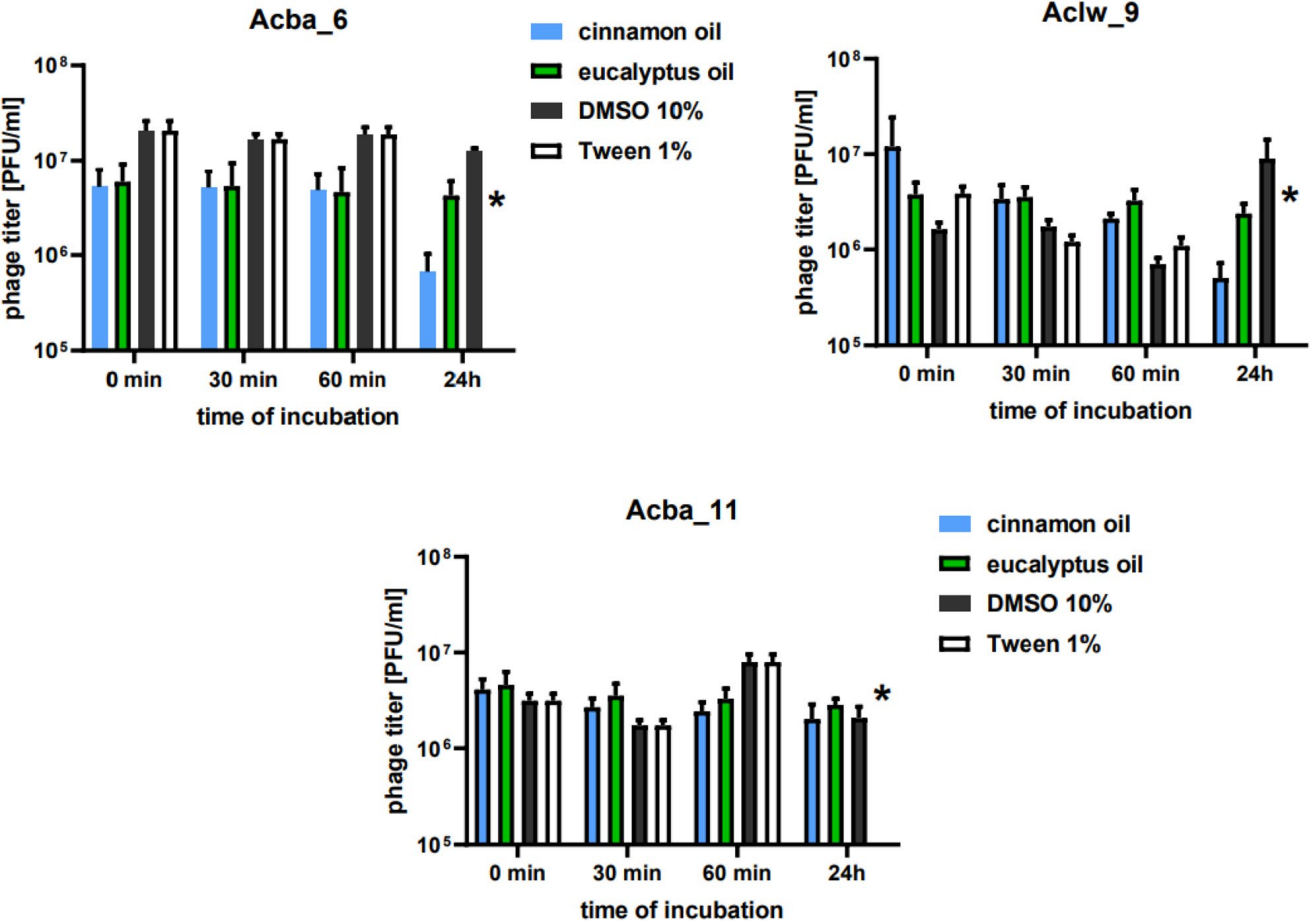
Stability of selected phages in cinnamon bark and eucalyptus essential oil, 10% DMSO and 1% Tween-20. Error bars represent the standard deviation (± SD) of the mean phage titers. *p < 0.05.

The largest decrease in the titer of phages incubated for 24 h in 1% cinnamon oil was noted for the phage Aclw_9—with a 1.6 order of magnitude (Fig. 5), while the smallest decrease in the titer after 24 h of incubation was noted for the phage Acba_13 and Acba_18—with a 0.1 order of magnitude. For 24 h of incubation of phages in 1% eucalyptus oil, the greatest decrease in titer was noted for phage Acba_3—with a 0.8 order of magnitude, and the smallest decrease in titer for phage Acba_13—with a 0.01 order of magnitude (Fig. S6). For 10% DMSO, the phage titer in Acba_1, Acba_15 and Acba_16 remain unchanged throughout the experiment. Acba_3, Acba_4, and Acba_14 had the most drastic decrease in phage titer at the end of the experiment, thus indicating the poorest stability in 10% DMSO. Acba_6 was also observed to have a decreased phage titer but unlike Acba_3, Acba_4, Acba_14, the decrease was not that visible. Surprisingly, Acba_18 had a slight increase in phage titer at the end of the experiment. It may be concluded that this bacteriophage can easily remain in 10% DMSO. As for the other phages, their titer fluctuates between measurement points but with the tendency to stay around the starting point. The plaques themselves were, in most cases, hardly visible and diminutive. As for the 1% Tween-20, a drastic decrease in phage titer was noticed in all tested phages except for Acba_14 where stability remained around 100% throughout the experiment. The phage titer of Acba_9 and Acba_3 gradually decreased, while for Acba_1, Acba_4, Acba_6, Acba_8, Acba_15, Acba_16 and Acba_18, the titers remain unchanged until the 24 h point, when they dropped drastically (Fig. 5, Fig. S6). The other phages showed slight fluctuations in phage titer with the tendency to decrease over time. Interestingly, it was observed that the addition of Tween 20 caused the plaques to become more visible and slightly bigger in comparison to those with 10% DMSO. Titers significantly differ only after 24 h incubation with Tween-20.

### Stability of phages in the urine

**Figure 6:**
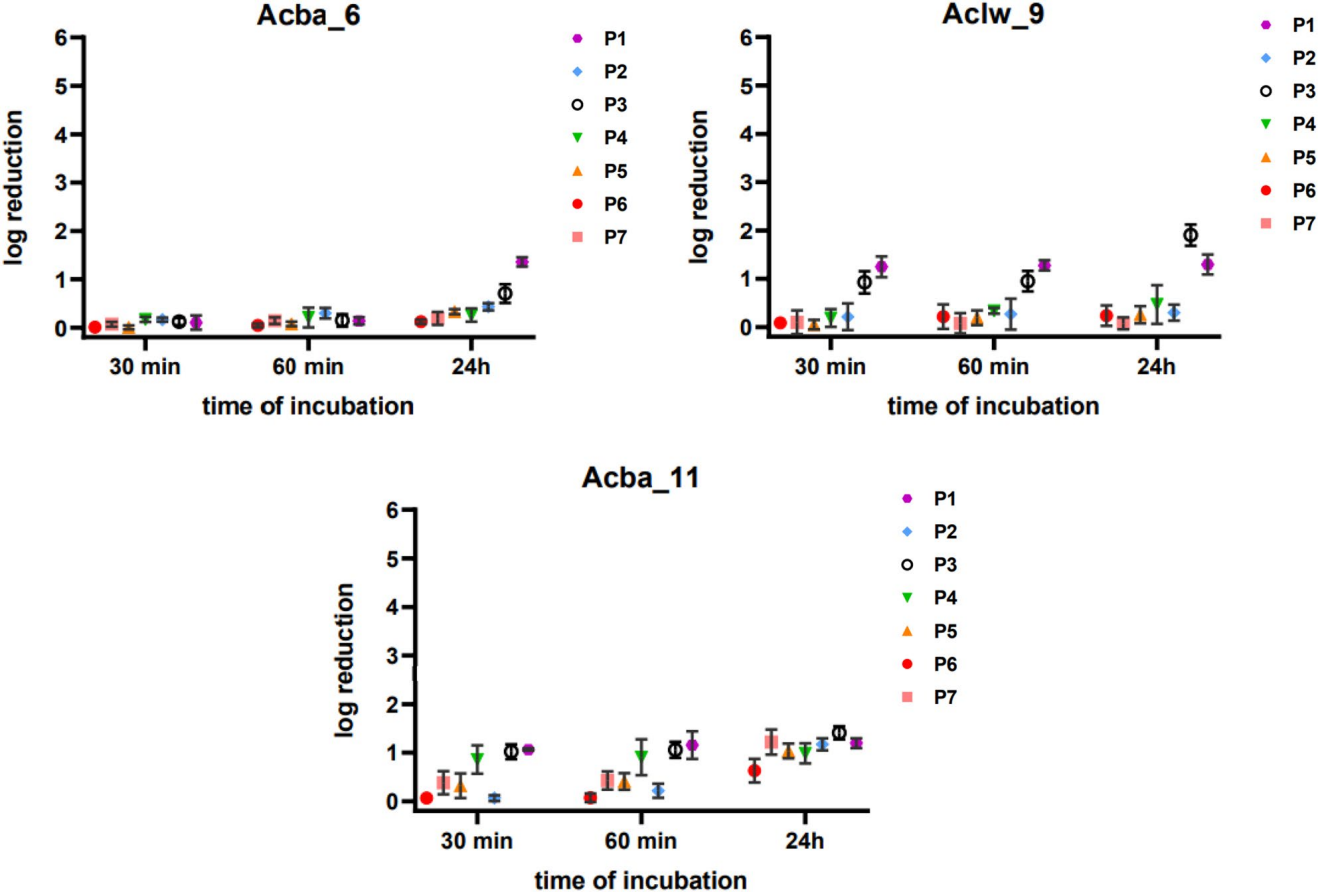
Stability of selected phages in urine during 30 min, 60 min and 24 h of incubation. Points corresponding to 0 min of incubation were used as a control. Error bars represent the standard deviation (± SD) of the mean phage titers.

The stability of phages in urine was tested in samples taken from both healthy individuals (n = 3) and from people with urinary tract infections (n = 4). In all urine samples (P1–P7) no abnormalities were found in the general urine test. However, in people with a UTI, the following bacteria were isolated from the urine: *E. coli* (P5, P6), *Klebsiella pneumoniae* (P4), *E. coli* and *K. pneumoniae* (P7). The greatest decrease in the titer of the Acba_6 phage was noted after 24 h of incubation in P1 urine by a 1.5 order of magnitude, while the smallest—after 24 h of incubation with P6 urine by a 0.1 order of magnitude (Fig. 6). The Aclw_9 phage was the most stable in P7 urine—its titer remained constant during 24 h of incubation. The highest decrease in titer was noted after 24 h of incubation in P3 and P1 urine by 1.8 and 1.3 orders of magnitude compared to the control. In turn, after 24 h of incubation in P2, P4, P5 and P6 urine, the Aclw_9 titer decreased by 0.2, 0.7, 0.5 and 0.4 orders of magnitude, respectively. After 24 h of incubation in urine P2, P3, P5, the phage titer decreased by 0.5 orders of magnitude, for sample P4 and P7 by 0.4 and 0.3 orders of magnitude, respectively. The Acba_11 phage turned out to be the most sensitive during 24 h of incubation in urine. A larger decrease in its titer was noted after incubation with P3 and P7 urine (by 1.4 orders of magnitude). Then, after 24 h of incubation in P1 and P2 urine, the titer decreased by 1.2 orders of magnitude; after incubation with P5, P4 and P6 urine, the Acba_11 titer decreased by 1.1, 1.0 and 0.5 orders of magnitude, respectively. The titer decreases are not statistically significant. Results for the remaining 9 bacteriophages are included in the supplement (Fig. S7).

### *A. baumannii* biofilm formation capacity and biofilm-reducing activity by phages

Based on the assessment of the biofilm-forming ability of *A. baumannii* strains isolated from urine, 6 strains were selected for further experiments with bacteriophages, 2 of which weakly (Ab2, Ab13), moderately (Ab10, Ab57) and strongly (Ab1, Ab68) produced biofilm. The biofilm produced by the Ab1 and Ab68 strains was sensitive to all phages/phage cocktail used. The biofilm of the Ab10 strain was insensitive to the Acba_1 and Aclw_9 phage, the biofilm produced by the Ab57 was insensitive to the 4 agents used, whereas the structure produced by the Ab2 strain was insensitive to the 8 agents. What is more, it was observed that the Ab13 strain was insensitive to all the agents used (regardless of whether single phages or a phage cocktail were used). The percentage of biofilm degradation (calculated by Eq. 1) by single bacteriophages and phage cocktail are presented in Table 3.

**Table 3:** Percentage of biofilm degradation by single bacteriophages and phage cocktail.

Phage/cocktail symbol	*A. baumannii* strain					
	Ab1	Ab2	Ab10	Ab13	Ab57	Ab68
	Percentage of biofilm degradation (%)					
Acba_1	62.4*	n.d.	n.d.	n.d.	n.d.	71.5*
Acba_3	85.6*	n.d.	23.4	n.d.	59.6	95.5*
Acba_4	82.6*	n.d.	47.4	n.d.	n.d.	77.0*
Acba_6	80.3*	41.5	51.7*	n.d.	33.9	89.5*
Aclw_8	92.7*	34.8	72.1*	n.d.	30.8	77.0*
Aclw_9	80.1*	n.d.	0.2	n.d.	44.6*	62.6
Acba_11	84.6*	29.2	68.6*	n.d.	62.3	65.8
Acba_13	78.6*	n.d.	5.3	n.d.	n.d	53.0*
Acba_14	83.0*	n.d.	11.3	n.d.	43.3	71.1*
Acba_15	81.7*	n.d.	24.8	n.d.	7.5	85.0*
Acba_16	87.1*	29.3	76.6*	n.d.	34.2	63.6*
Acba_18	95.0*	53.7*	92.5*	n.d.	23.9	84.4*
K1 (Acba_6; Acba_9; Acba_11)	73.7*	n.d.	24.0	n.d.	n.d.	52.9*

*n.d.* no biofilm degradation.

*Statistically significant results, p < 0.05.

p-value is calculated between three technical replicates and expected.

value of 0% for non-degraded biofilm (GraphPad Prism).

## Discussion

In this paper, we presented results regarding the stability of *A. baumannii*-specific phages under different storage conditions: temperatures with or without glycerol added, different pH values, disinfectants, metal nanoparticles, essential oils, surfactants as well as urine. On the one hand, our research will allow us to better understand the possibility of ensuring storage conditions in which the phage preparations containing the studied phages do not lose their activity. On the other hand, examination of the activity in the presence of selected disinfectants, solutions of nanoparticles with known antibacterial activity or natural compounds (essential oils) will allow us to determine which of these agents could potentially support the antibacterial activity of these phages in preparations used in the fight against UTIs. Moreover, testing their stability in the urine of patients will allow us to determine whether such urine may have an inactivating effect on them, which may give us an idea of the possibility of A. baumannii phages retaining antibacterial activity at the site of infection, in the bladder. Recent studies on the stability of therapeutic phages have shown that this issue should be approached whilst also taking into account the method of administration of the phage preparation^36^. Although data in the literature suggest the effectiveness of using A. baumannii phages in combination with antibiotics^37,38^, the results presented in this paper point towards the possibility of using phages combined with nanoparticles, or essential oils as well. Studies on phages specific for *A. baumannii* in the future may create an opportunity to use these phages in the treatment of infections caused by MDR strains of *A. baumannii*.

Both from our experience and based on other scientists’ observations^39^, it is known that the use of phages for therapeutic purposes necessitates a search for ways to maintain a stable high titer both during the storage process and after administration of the phage preparation. Available data indicate that phages can be inactivated by various factors. Studies show that exposure to low or high temperatures leads to the inactivation of, e.g., the MS2 phage^40^ and P680 phage^41^, respectively. In turn, phage P001^42^ is sensitive to changes in pressure. Radiation—infrared and microwaves—leads to inactivation of phage Φ6^43^ and T4, T7, λ, MS2 phages, respectively. Another physical method which causes a loss in titer in the case of MS2 and M13 is the use of columns of microplastic^44^. In addition to the methods mentioned above, factors leading to the inactivation of phages may include: ozone, UV, ultrasound, electric field, plasma, humidity, visible light or energetic femtosecond lasers. Besides the physical factors, chemical substances and factors can also cause damage to phage virions. Therefore, taking into account the therapeutic potential of phages, it is important to try to assess what factors may affect them and examine their effect on different phages. Despite the fact that many factors act on phages simultaneously in the human body, it would unfortunately be impossible to take them all into account at once. Therefore, the observed effect of individual factors on phages may be different from that observed under storage conditions or after administration of a therapeutic preparation, and we are aware that this may be one of the limitations of our research. Another was the attempt to determine the titer of phages in disinfectants that have a destructive effect on bacteria, and therefore, when inhibiting bacterial growth, we had difficulty in determining the titer of phages. What is more, achieving and maintaining a high titer of *Acinetobacter*-specific phages is extremely difficult compared to other bacteria, such as *E. coli* or *Enterobacter cloacae*. Some of the tested clinical strains of *A. baumannii* were characterized by unusual growth.

The long-term (1 year) temperature stability of phages was tested under the following conditions: at 4 °C, room temperature, − 70 °C and − 70 °C with the addition of 25% glycerol. Phage activity (in lysate form) decreased the fastest at room temperature. In the case of the Aclw_9 phage (Fig. 2) and the Acba_16 phage (Fig. S1), after 2 months of incubation at room temperature, the phage preparation almost completely lost its activity. The most optimal storage conditions for phages turned out to be − 70 °C both with and without the addition of 25% glycerol (Fig. 2, Fig. S1). In addition, the stability of the phages at 60 °C for 1 h of incubation was tested. The smallest decrease in titer was noted for the Acba_4 phage by 2.6 orders of magnitude and the largest for Acba_1 by 4.5 orders of magnitude as well as Acba_6, Aclw_8 by 4.4 orders of magnitude (Fig. 3, Fig. S2). Jiang et al. conducted stability studies of the lytic phage Abp9 (specific for clinical *A. baumannii* isolate ABZY9) at different temperatures (20, 30, 40, 50, 60, and 70 °C) for 15 min^45^. This phage was stable at a temperature range of 20–50 °C, but at 60 °C its titer decreased 10 times after 15 min of incubation, and at 70 °C only 1% of the active phages were detected after 15 min of incubation. Soontarach et al. also conducted a study on the temperature stability of *A. baumannii*-specific phages isolated from hospital wastewater in Thailand^46^. After 2 h of incubation at temperatures from − 20 to 60 °C, most of the 12 tested phages turned out to be stable, with only phage P1033 losing more than 50% of its activity after incubation at 60 °C, while none of the phages showed activity after incubation at 70 °C after 2 h of incubation. In addition, phage titers were stable for 2 years of incubation at 4 °C in SM buffer. The study of long-term storage of phages in various temperatures in the presence of various agents (e.g., 20% glycerol, 0.05% sodium azide, 10% DMSO + 5% chloroform) contributes to the development of phage therapy^47^.

Research on the stability of phages at different pH shows that the most optimal solution for storing phages is one with a neutral or slightly alkaline reaction (pH from 7.0 to 9.0). Because phages are structures consisting of proteins, these proteins can be denatured at a low pH^48^. It was noted that a low pH of 3.0–4.0 significantly affects the loss of phage activity, while even a strong alkaline reaction (pH 11.0–12.0) does not lead to a complete loss of phage activity (Fig. 4, Fig. S3). Other studies on *A. baumannii*-specific phages show that these phages were stable at pH 5.0 to 9.0—with most retaining 80% activity over 2 h of incubation. Only the P1033 phage turned out to be less stable with less than 48% active in a solution of pH 9.0^46^. Additionally, the Abp9 phage is stable at different pH (pH 7.0–11.0). A significant decrease in the titer for this phage was noted for pH from 3.0 to 6.0 and from 11.0 to 12.0^45^.

Phage stability was also tested in commonly used disinfectants. The most effective in phage inactivation was agent 2, after incubation with which no active phage particles were detected. Ethyl alcohol-based disinfectants (agent 3, agent 4) as well as the 70% ethanol solution alone led to rapid phage inactivation. Agent 1 turned out to be an equally effective phage inactivating agent (Table 2). Interesting studies on the stability of phages, including those of *A. baumannii* (Acibel004, Acibel007) and phages specific for *P. aeruginosa* and *S. aureus* were carried out by Merabishvili et al.’s group, in which phage stability was tested in 13 various burn wound care products^49^. The most stable phage turned out to be phage 14-1, active against *P. aeruginosa*—its titer in the presence of most of the agents was at a level of 10^7^ PFU/mL during 4 h of incubation. The remaining 4 phages showed similar stability, and their greatest inactivation was caused by anti-infective products, such as Bactroban, iso-Betadine, P.O.H. ointment and colistin milk. In addition, agents with high acidity had the most adverse effect on phage activity (this observation is in accordance with our results in pH experiments). On the other hand, another phage specific for *A. baumannii* (AbTJ phage) was tested for its stability in the presence of isopropanol and ethanol of various concentrations. The most effective in reducing the phage titer turned out to be 100% ethanol and 95% isopropanol, which after 90 min reduced the phage titer by 82% and 91%, respectively^50^.

Phage stability in silver or copper nanoparticle solutions varied between phages, but phage titers remained constant for 30 min of incubation (Table 2). Interestingly, colloidal silver can be used as an antibacterial agent. Green synthesized colloidal silver (GSCS) has been shown to have an anti-biofilm effect on clinical isolates of *P. aeruginosa*, *S. aureus* and *Haemophilus infuenzae*^51,52^. In addition, it has been observed that GSCS has an antibacterial effect not only on biofilm but also on the planktonic form of *Mycobacterium abscessus*, albeit with a greater effectiveness against the planktonic form of the bacteria^53^. Colloidal silver and silver ions interact with lipids, proteins and lipopolysaccharides in the biofilm matrix, which results in damage to the bacterial biofilm^54^. In addition to silver-containing colloids, copper colloids also have a bactericidal effect. Studies using copper nanoparticles (CuNPs) showed their bactericidal activity against *E. coli* (MTCC no. 739) and *Proteus vulgaris* (MTCC no. 426) strains. CuNPs caused loss of bacterial membrane permeability, leakage of cytoplasmic components and production of reactive oxygen species (ROS) which resulted in bacterial cell death. Interestingly, greater cytoplasmic leakage and faster growth inhibition were noted for *E. coli* compared to *P. vulgaris*^55^. In addition, a bactericidal or bacteriostatic effect was demonstrated for copper alloys (CuZn37, CuSn6 and CuNi18Zn20), including copper Electrolytic Tough Pitch (ETP) (99.9% Cu) against clinical strains of *A. baumannii* and *Acinetobacter lwoffii* and *A. pittii* isolated from hospital environments. The best killing activity against the *Acinetobacter* strains used in the study (the fastest and the largest reduction of the optical density of the bacterial suspension) was noted for ETP copper^56^. Interestingly, recent research has shown a synergistic effect of the ZCSE6 phage and green synthesized zinc oxide nanoparticles (ZnO-NPs) of *Ocimum basilicum* extract using the bio-reduction process. ZnO-NPs have been shown to have a reducing effect on *Staphylococcus sciuri* biofilm. In contrast, combining the aforementioned phage and ZnO-NPs caused them to act synergistically in the fight against *Salmonella enterica*^57^. In addition, other studies on nanoparticles have shown that using the cyclic 9-amino acid peptide CARGGLKSC (CARG), identified via phage display on *S. aureus*, increases the effectiveness of therapy with the use of CARG-conjugated vancomycin-loaded nanoparticles. In mice with *S. aureus*-induced pneumonia, the peptide selectively binds to bacteria in infected lung tissue and thus increases the concentration of intravenously injected vancomycin-loaded porous silicon nanoparticles at the site of infection^58^. Metal nanoparticles, e.g., gold nanoparticles, can also cause phage inactivation, while having a protective effect on bacteria, which may be of key importance from the point of view of biotechnology and industry^59^.

An interesting issue is the effect of essential oils on bacteria, for example, in the study on *Litsea cubeba* essential oil (LCEO) against *A. baumannii*. Scanning electron microscopy (SEM) and sodium dodecyl sulfate polyacrylamide gel electrophoresis (SDS-PAGE) studies confirmed changes in bacterial cell membrane integrity and permeability as well as leakage of intracellular biomacromolecules. Microscopic imaging showed changes in the morphology of bacterial cells after applying the oil. Additionally, LCEO was also found to cause changes in the expression of bacterial proteins^60^. In contrast, the use of thymol and/or eugenol derived from essential oils in combination with endolysin LysECP26 resulted in a four-fold decrease in the minimum inhibitory concentration of LysECP26 for *E. coli* O157:H7 compared to the use of agents alone. Moreover, SEM studies showed disruption of the bacterial cell wall and their morphological changes after the use of endolysin in combination with thymol and/or eugenol^61^. In our study, we investigated the effect of two essential oils (cinnamon and eucalyptus) on the stability of 12 *A. baumannii*-specific phages (Fig. 5, Fig. S6). For cinnamon oil, the smallest decrease in titer (compared to time zero) after 24 h of incubation was observed for phage Acba_13 and Acba_18 by a 0.1 order of magnitude, while the largest decrease in titer for phage Aclw_9 by a 1.6 order of magnitude. In the case of eucalyptus oil, the smallest decrease in titer (compared to time zero) after 24 h of incubation was observed for phage Acba_13 and Acba_18 by a 0.01 order of magnitude, while the largest decrease in titer for phage Acba_3 was a 0.8 order of magnitude. Decreases in phage titers after incubation in essential oils were not statistically significant. Bacteriophage titer was also tested in different surfactants. For this experiment we chose and tested phage stability in 10% DMSO and in 1% Tween-20. The phages tend to better tolerate 10% DMSO than 1% Tween-20 solution. More bacteriophages remain at their starting point titer in 10% DMSO. Additionally, their stability, for all 12, 10% DMSO cases, did not drop as drastically as in the 1% Tween-20 solution. Surprisingly the plaques themselves were larger and more visible in 1% Tween-20 in comparison to 10% DMSO. Available data in the literature show that organic solvents such as DMSO, tetrahydrofuran, isopropanol, and ethanol can be used as a carrier for bacteriophages: *P. aeruginosa*-specific myovirus (PEV1, PEV20) and podovirus (PEV31) phages. However, it is optimal to use a concentration below 50% (v/v). These reports are promising for the formulation of pharmaceuticals containing phages^62^.

The presence of phages (both lytic and temperate ones) was confirmed in the urinary tract^63–65^. Although the role of phages in the urinary tract is still unknown, it has been suggested that they may influence urinary health^65^. It may be beneficial to emphasize phage stability in urine when it is intended to administer them locally, e.g., intravesically in the treatment of UTIs^66^. The obtained results were intended to verify whether phages that could be used in the future to treat urinary tract infections are able to retain the ability to specifically destroy bacteria after incubation in urine with a pathological composition. In our experiments, phage incubation in human urine indicated that their titer remained at a similar level for 60 min of incubation, and a decrease in the titer (statistically insignificant) could be seen after 24 h (Fig. 6, Fig. S7) regardless of whether the urine sample came from a healthy person or a patient with a diagnosed UTI. We detected no statistically significant correlation (Pearson) between the measured decrease in phage titers after 24 h and urine specific gravity for any tested phage (data not shown). The stability of phages during the first hour of incubation may be important in the future in the treatment of UTIs using phage therapy. Urinary stability studies of healthy individuals with phages (Entb_43, Entb_45) specific for *Enterobacter* strains confirm the stability of the phages for the first hour of incubation in urine. But therapeutic phage OMKO1 specific for *P. aeruginosa* showed stability in various concentrations of saline, even in 5 M saline for 90 min of incubation. 3 M and 4 M urea solutions caused a significant decrease in phage titer after 90 min of incubation^67^. This is a prime example for why it is so important to indicate under what conditions individual phages can maintain their therapeutic value.

An important virulence feature among *A. baumannii* strains is the ability to form a biofilm. Interestingly, studies have shown that *Vibrio cholerae* are able to form a biofilm on human macrophages. These bacteria express and secrete the high concentrations of hemolysin HlyA at the site of biofilm production, resulting in the killing of the immune cells*. V. cholerae* lacking the ability to form a biofilm and the strain lacking the gene encoding hemolysin HlyA caused macrophage death to a lesser extent than wild strains^68^. The above example shows how difficult and important it is to fight the biofilm produced by pathogens. Studies on the effect of disinfectants on *A. baumannii* indicate that biofilm is less sensitive to disinfectants than planktonic bacteria^69^. Planktonic MDR clinical strains of *A. baumannii* exposed to bleach, ethanol, quaternary ammonium compounds, chlorhexidine gluconate, and povidone were 99.9% eradicated after 4 min of incubation. In contrast, for biofilm, survival was 6.23% after 1 min with 55% ethanol, 3.7% after 4 min with bleach, and 3.3% after 1 min with 70% ethanol.

Based on the presented results of biofilm degradation of uropathogenic *A. baumannii* strains by phages/cocktails, it can be concluded that single phages work better than phage cocktails, which may be surprising. The latest research confirms the effectiveness of phage cocktails against MDR bacteria. This phage cocktail, consisting of 5 prophages (2 A. baumannii prophages and 3 K. pneumoniae prophages), was computationally developed and was effective in lysing carbapenemase-producing strains, both identified as *Enterobacter hormaechei* and *K. pneumoniae* strains^70^. However, according to the literature, the use of single phages may result in the emergence of resistance among bacterial strains to the phage. This problem can be solved by using phage cocktails^71,72^. In addition, in preliminary tests of the sensitivity of MDR bacterial strains to phages (phage typing), both sensitive (Ab1, Ab2, Ab10) and insensitive strains (Ab13, Ab57, Ab68) were selected. The biofilm of the Ab1 strain was sensitive to all the phages used, in contrast to the biofilm of the Ab2 and Ab10 strains, where some phages did not have a degrading effect on the formed biofilm. However, in the abovementioned biofilm degradation studies, the biofilm of the Ab13 strain was found to be insensitive to the action of phages, however, the biofilm of the strains Ab57 and Ab68 turned out to be sensitive to the action of 9 phages and all phages, respectively (Table 3). This may be due to the fact that during the study of biofilm degradation, bacterial biofilm was incubated for 24 h in the presence of 200 µL of the phage/phage cocktail in a well of a 96-well plate, which could have contributed to the better action of phages on the biofilm of uropathogenic strains. Interestingly, a study on biofilm produced by *E. coli* strains (DS515 and DS552) isolated from the urine of patients with a spinal cord injury showed that 9 phages (HP3, ES17, 6950, 6915, 6955, HP3.1, 6935, ES21, ES19) of the 28 tested reduced cell viability by more than 80% with a single application to the biofilm of both bacterial strains in human urine. In addition, the use of a phage cocktail (HP3, ES12, ES17, ES19, ES21, ES26) against young and old biofilms and biofilms on silicone catheter materials confirmed its effectiveness against *E. coli* biofilm. Moreover, this cocktail lysed 82% of the pool of 54 *E. coli* strains isolated from the urine^73^. Interestingly Tapia-Rodriguez et al*.* observed that oregano essential oils as well as terpene compounds carvacrol and thymol showed an ability to inhibit *A. baumannii* biofilm development and indicated that carvacrol reduced the structure even in 95% at concentration 0.15 mg/mL^74^. The abovementioned studies indicate that there is a possibility to also use essential oils in combating biofilm produced by *A. baumannii* strains.

Interestingly, thanks to the development of artificial intelligence, scientists were able to select 9 compounds from a group of almost 7000, and one of these turned out to be particularly effective in combating *A. baumannii*^75^. The above examples show that the development of artificial intelligence can significantly influence the development of therapies aimed at bacteria resistant to currently used drugs. Despite there being a recently new class of antibiotics active against isolates of CRAB both in vitro and in vivo^76,77^, this will not result in the abandonment of research on bacteriophages and possibilities for their therapeutic use.

## Conclusion

The presented stability studies, and in particular studies of phage stability in urine, may have a fundamental impact on the development of phage therapy application in urinary tract infections caused by MDR strains of *A. baumannii*. This is especially the case as phage therapy is now a serious alternative to widely used antibiotic therapy.

## Future perspective

In the next paper we also plan to present the potential of the activity of a combination of described phages with different agents in degrading *A. baumannii* biofilm on the catheter surface as was described by Oleksy-Wawrzyniak et al. on biofilm produced by *K. pneumoniae*^78^. This will shed new light on the possibility of using phages in combination with natural ingredients with proven antibacterial activity in the fight against urinary tract infections caused by such dangerous pathogens as *A. baumannii*.

## Materials and methods

### Bacterial strains and bacteriophages

There were 257 strains belonging to *A. baumannii* used in the study, which were obtained in 2018–2022 as part of a cooperation with Polish hospitals as described elsewhere. Ten of them (Table 1) were used for biofilm research, and the rest were used to search for bacteriophages specific to *A. baumannii* as well as to determine the biological properties of isolated phages^34^. Antibiotic susceptibility was determined by the MicroScan WalkAway analyzer (Beckman Coulter, Brea, CA, USA). Species affiliation was determined by matrix-assisted laser desorption/ionization—time of flight (MALDI-TOF/TOF) mass spectrometry (Bruker Daltonics, Billerica, MA, USA). All bacterial strains were stored at − 70 °C in 25% glycerol. After thawing, the strains were cultured on plates with McConkey agar (peptone 17 g, proteose peptone 3 g, lactose 10 g, bile salts 1.5 g, sodium chloride 5 g, neutral red 0.03 g, crystal violet 0.001 g, agar 13.5 g, water—added to make 1 L; and pH adjusted to 7.1 ± 0.2 sodium taurocholate) at 37 °C for 12 h. During the studies, 12 bacteriophages specific for *A. baumannii* were isolated as a result of screening 460 water samples. Bacteriophages were isolated, amplified and their biological properties were as described previously^34^.

### Bacteriophage amplification

Prior to the stability study, all phage lysates were amplified to obtain the highest possible titer in order to observe its potential decrease while testing the stability of *A. baumannii*-specific bacteriophages. Up to 50 mL peptone water (per 1000 mL of water: meat extract 0.40 g, yeast hydrolysate 1.70 g, NaCl 3.50 g, Bacto Peptone 4.00 g enzymatic hydrolyzate of casein 5.40 g) liquid 2-h bacterial culture and phages were added in such a ratio so as to obtain the optimal MOI^34,66^. After overnight incubation at 37 °C, the samples were filtered (with a pore diameter of 0.22 µm, Millipore, Burlington, MA, USA) and Routine Test Dilution (RTD) was performed. For this purpose, on a previously prepared plate with a bacterial lawn on the solidified agar surface, drops of 50 µL serial dilutions of the filtered samples (10^0^–10^–8^) were applied. The plates were pre-dried at room temperature, incubated overnight at 37 °C, and then the plaques were counted. The double-layer agar method was performed to determine the titer of the obtained phages^79,80^. A culture of the appropriate bacterial strain (100 µL) and appropriate phage dilution were added to 2 mL of melted 0.7% agar. The agar with bacteria and phages (200 µL) was mixed and poured onto a solid agar plate. Plates were left for a few minutes at room temperature, incubated overnight at 37 °C, and the titer of bacteriophages was subsequently calculated.

### Phage stability under various temperature conditions

Phage lysates with an initial titer of ~ 10^7^ PFU/mL were stored in various temperature conditions: 60 °C, 4 °C, room temperature (23 °C), − 70 °C and − 70 °C with the addition of 25% glycerol (Difco). Samples incubated at 60 °C (water bath) were taken every 10 min for an hour. In other cases, phage titer was checked for 12 months (samples were collected once a month). The experiment was performed in triplicate. For titer determination the RTD and the double-layer agar method was performed as described above.

### Phage stability under various pH values

In order to test the stability of bacteriophages at different pH values, solutions of peptone water with a pH ranging from 3 to 12 were prepared (established with 5 M HCl or 10 M NaOH). A solution with a pH of 7.0 (standard culture medium) served as a control sample. Phage lysates (titer ~ 10^7^ PFU/mL) were added to the samples at a ratio of 1:9 (phage: peptone water). The samples prepared in this way were incubated at 37 °C. Then, they were taken after 1 and 24 h of incubation. The experiment was performed in triplicate. The serial dilutions were prepared for each sample and the phage titer was also evaluated.

### Phage stability in various disinfectants and metal ions

The stability of bacteriophages was also tested in selected detergents and disinfectants: 70% ethanol, 10% agent containing aluminum hydrochloride (agent 1), agent containing octenidine dihydrochloride and phenoxyethanol (agent 2), disinfectant containing ethanol (57 g/100 g) and propane 2-ol (6 g/100 g) (agent 3), as well asdisinfectant containing ethanol (70 g/100 g) (agent 4). Additionally, copper and silver nanoparticle solutions (Nano-Tech, Poland) at a concentration of 50 mg/kg (ppm) were used during the stability study. Phage lysates with the mentioned agents were incubated at room temperature at a ratio of 1:9. Samples were taken after 5, 10, 20 and 30 min of incubation, except for samples containing Bactcid AF and Hysepta AV where samples were taken already after 30 and 60 s of incubation^66,80^. Serial dilutions were made from each sample and the phage titer was checked. The experiment was performed in triplicate.

### Phage stability in chosen essential oils and surfactants

Phage stability was also tested in the presence of 1% cinnamon bark oil and eucalyptus essential oil (CIN2005 and EUC2B01, Essence, Konstancin-Jeziorna). Phages (titer ~ 10^7^ PFU/mL) and 1% essential oil suspension (in the presence of a 10% of dimethyl sulfoxide (DMSO) and 1% of Tween-20) were mixed at a ratio of 1:9 and incubated at 37 °C. The samples were taken after 30 min, 1 h and 24 h of incubation. The serial dilutions were made and the phage titer was determined with the use of the RTD and the double-layer agar method. The experiment was performed in triplicate. In addition, the effect of DMSO and Tween-20 on phage stability was also investigated (similarly to that described above).

### Stability of phages in the urine of patients with a urinary tract infection (UTI)

Urine (midstream) was collected from both patients diagnosed with a urinary tract infection (UTI) n = 4 and healthy donors n = 3. Urine samples for testing were collected from women (P3, P4, P6, P7) and men (P1, P2, P5). To assess the physicochemical parameters of each sample the urine sediment test was performed. Urine samples were filtered through syringe filters with a pore diameter of 0.22 µm (Millipore, Burlington, MA, USA). Phage lysates (titer ~ 10^7^ PFU/mL) and urine samples were mixed at a ratio of 1:9 and incubated at 37 °C. Phage titers at time zero of the experiment were taken as controls. The experiment was performed in triplicate. After 30 min, 1 h and 24 h the serial dilutions were made and the phage titer in the tested urine was determined^66^.

### Evaluation of biofilm formation capacity and phage activity in biofilm eradication

Ten uropathogenic multidrug-resistant clinical strains of *A. baumannii* isolated from urine were selected for biofilm studies based on previous phage typing results: 5 sensitive (Ab1, Ab2, Ab3, Ab10 and Ab12) and 5 insensitive (Ab13, Ab39, Ab57, Ab68 and Ab82) to the 12 studied phages. The method of obtaining biofilm of *A. baumannii* strains was developed by Grygorcewicz et al. with some modifications^81^. Overnight bacterial cultures (incubated at 37 °C) of these strains were prepared in sugar broth (in the composition: meat extract 0.4 g, enzymatic hydrolysate of casein 5.4 g, yeast hydrolysate 1.7 g, Bacto Peptone 4.0 g, NaCl 3.5 g, glucose 10 g per 1000 mL of H_2_O). Next, the optical density of the obtained bacterial cultures at wavelength λ = 600 nm (OD_600_) was measured spectrophotometrically (BioSpectrometer basic, Eppendorf, Hamburg, Germany). These suspensions were then diluted to the following optical densities: 0.1, 0.2, and 0.5. Then, 200 µL per well of the appropriate dilution of each strain was applied to a 96-well plate and incubated for 48 h at 37 °C. Next, the plates were washed twice with phosphate-buffered saline (PBS) and dried at 60 °C for 30 min. Then 200 µL per well of 1% crystal violet (Chempur, Piekary Śląskie, Poland) was added and incubated for 30 min. After incubation, the wells were washed with water, then 95% ethanol was applied to the wells and incubated for 30 min. Bacterial cells were stained with ethanol. The quantification of the washed crystal violet is proportional to the number of bacterial cells forming the biofilm. The absorbance at a wavelength of λ = 570 nm was measured. The ability to form a biofilm was estimated based on the scheme proposed by Stepanović et al.: OD ≤ ODc—strain does not produce biofilm; ODc < OD ≤ 2ODc—strain weakly produces biofilm; 2ODc < OD ≤ 4ODc—strain moderately produces biofilm; 4ODc < OD strain strongly produces biofilm. OD is the optical density of the tested sample, ODc is the optical density of blank sample measured at a wavelength of λ = 570 nm^82^. To assess the ability of phages/phage cocktails to reduce bacterial biofilm, a 48-h biofilm was washed twice with PBS and phage lysates/phage cocktails were applied to the wells with the formed biofilm. The plates were incubated for 24 h at 37 °C. The next steps of the procedure were the same as described above. The study used single phages in the form of phage lysates and a phage cocktail with the following composition: K1 (Acba_6 + Acba_9 + Acba_11). Phages with the widest lytic spectrum were used to prepare phage cocktails. The experiment was performed in triplicate. The percentage of biofilm degradation by bacteriophages was assessed using the Eq. (1) below^83^.

1 $$deg\%=\left(1-\frac{{OD}_{treated}}{{OD}_{untreated}}\right)\times 100\%.$$

### Statistical analysis

A one-way ANOVA was used to assess changes in phage titer throughout incubation time. Statistical significance was determined using Dunnett’s multiple comparison test (GraphPad Prism 2023). The one-sample t-test and the Wilcoxon test using GraphPad, version 9.5.1 (2023) were used to study the stability of phages in various disinfectants and solutions of copper and silver. Statistical significance was considered at p < 0.05.

### Ethical approval

Experiments using human urine samples entitled “Study of phage stability in the urine of patients diagnosed with urinary tract infection” were approved by the Bioethics Committee at the Ludwik Hirszfeld Institute of Immunology and Experimental Therapy Polish Academy of Sciences, consent number: KB-12/2022. All methods were performed in accordance with the relevant guidelines and regulations. Each patient signed an informed consent form and was informed about the purpose for which the collected urine would be used.

### Supplementary Information

Supplementary Figures.

## Data Availability

All data generated or analysed during this study are included in this published article (and its Supplementary Information files).

## References

[1] Kuehn M (2022). Progress against antimicrobial resistance has slipped. JAMA.

[2] Mączyńska B (2023). Changes in antibiotic resistance of *Acinetobacter baumannii* and *Pseudomonas aeruginosa* clinical isolates in a multi-profile hospital in years 2017–2022 in Wroclaw, Poland. J. Clin. Med..

[3] World Health Organization (WHO). http://www.who.int/mediacentre/news/releases/2017/bacteria-antibiotics-needed/en (2018).

[4] World Health Organization (WHO). https://www.who.int/emergencies/ten-threats-to-global-health-in-2019 (2019).

[5] Lin MF, Lan CY (2014). Antimicrobial resistance in *Acinetobacter baumannii*: From bench to bedside. World J. Clin. Cases.

[6] Lee CR (2017). Biology of *Acinetobacter baumannii:* Pathogenesis, antibiotic resistance mechanisms, and prospective treatment options. Front. Cell Infect. Microbiol..

[7] Fournier PE, Richet H (2006). The epidemiology and control of *Acinetobacter baumannii* in health care facilities. Clin. Infect. Dis..

[8] Jawad A, Heritage J, Snelling AM, Gascoyne-Binzi DM, Hawkey PM (1996). Influence of relative humidity and suspending menstrua on survival of *Acinetobacter* spp. on dry surfaces. J. Clin. Microbiol..

[9] Towner KJ (2009). *Acinetobacter:* An old friend, but a new enemy. J. Hosp. Infect..

[10] Peleg AY, Seifert H, Paterson DL (2008). *Acinetobacter baumannii*: Emergence of a successful pathogen. Clin. Microbiol. Rev..

[11] Maragakis LL, Perl TM (2008). *Acinetobacter baumannii*: Epidemiology, antimicrobial resistance, and treatment options. Clin. Infect. Dis..

[12] Perez F (2007). Global challenge of multidrug-resistant *Acinetobacter baumannii*. Antimicrob. Agents Chemother..

[13] Jerassy Z (2006). Prospective hospital-wide studies of 505 patients with nosocomial bacteraemia in 1997 and 2002. J. Hosp. Infect..

[14] Robenshtok E (2006). The significance of *Acinetobacter baumannii* bacteraemia compared with *Klebsiella pneumoniae* bacteraemia: Risk factors and outcomes. J. Hosp. Infect..

[15] Martinez-Guitián M (2021). Global transcriptomic analysis during murine pneumonia infection reveals new virulence factors in *Acinetobacter baumannii*. J. Infect. Dis..

[16] Harding CM, Hennon SW, Feldman MF (2018). Uncovering the mechanisms of *Acinetobacter baumannii* virulence. Nat. Rev. Microbiol..

[17] Eijkelkamp BA, Stroeher UH, Hassan KA, Paulsen IT, Brown MH (2014). Comparative analysis of surface-exposed virulence factors of *Acinetobacter baumannii*. BMC Genom..

[18] Ramirez MS (2019). Identification of potential virulence factors in the model strain *Acinetobacter baumannii* A118. Front. Microbiol..

[19] Bai J, Raustad N, Denoncourt J, van Opijnen T, Geisinger E (2023). Genome-wide phage susceptibility analysis in *Acinetobacter baumannii* reveals capsule modulation strategies that determine phage infectivity. PLoS Pathog..

[20] Vázquez-López R (2020). *Acinetobacter baumannii* resistance: A real challenge for clinicians. Antibiotics (Basel).

[21] Gerson S (2019). Investigation of novel pmrB and eptA mutations in isogenic *Acinetobacter baumannii* isolates associated with colistin resistance and increased virulence in vivo. Antimicrob. Agents Chemother..

[22] Cai Y, Chai D, Wang R, Liang B, Bai N (2012). Colistin resistance of *Acinetobacter baumannii:* Clinical reports, mechanisms and antimicrobial strategies. J. Antimicrob. Chemother..

[23] Pormohammad A (2020). Global prevalence of colistin resistance in clinical isolates of *Acinetobacter baumannii*: A systematic review and meta-analysis. Microb. Pathog..

[24] Pakharukova N (2018). Structural basis for *Acinetobacter baumannii* biofilm formation. Proc. Natl. Acad. Sci. U.S.A..

[25] Pour NK (2011). Biofilm formation by *Acinetobacter baumannii* strains isolated from urinary tract infection and urinary catheters. FEMS Immunol. Med. Microbiol..

[26] Narayanan A (2016). Inactivation of *Acinetobacter baumannii* biofilms on polystyrene, stainless steel, and urinary catheters by octenidine dihydrochloride. Front. Microbiol..

[27] Gliźniewicz M (2024). Advances in bacteriophage-mediated strategies for combating polymicrobial biofilms. Front. Microbiol..

[28] Li Y, Xiao S, Huang G (2023). *Acinetobacter baumannii* bacteriophage: Progress in isolation, genome sequencing, preclinical research, and clinical application. Curr. Microbiol..

[29] Wintachai P, Surachat K, Chaimaha G, Septama AW, Smith DR (2022). Isolation and characterization of a *Phapecoctavirus* infecting multidrug-resistant *Acinetobacter baumannii* in A549 alveolar epithelial cells. Viruses.

[30] Huang L (2023). Characterisation and sequencing of the novel phage Abp95, which is effective against multi-genotypes of carbapenem-resistant *Acinetobacter baumannii*. Sci. Rep..

[31] Chen LK (2022). Preoptimized phage cocktail for use in aerosols against nosocomial transmission of carbapenem-resistant *Acinetobacter baumannii:* A 3-year prospective intervention study. Ecotoxicol. Environ. Saf..

[32] Narulita E (2024). Bacteriophage ɸPT1b-based hand sanitizer gel for reducing pathogenic *Escherichia coli* infection. Indian J. Microbiol..

[33] Kim K (2021). Characterization of a novel phage ΦAb1656-2 and its endolysin with higher antimicrobial activity against multidrug-resistant *Acinetobacter baumannii*. Viruses.

[34] Bagińska N (2023). Biological properties of 12 newly isolated *Acinetobacter baumannii*-specific bacteriophages. Viruses.

[35] Ghajavand H (2017). Isolation of bacteriophages against multidrug resistant *Acinetobacter baumannii*. Res. Pharm. Sci..

[36] Uyttebroek S (2023). Stability of magistral phage preparations before therapeutic application in patients with chronic rhinosinusitis, sepsis, pulmonary, and musculoskeletal infections. Microbiol. Spectr..

[37] Schooley, R. T. *et al*. Development and use of personalized bacteriophage-based therapeutic cocktails to treat a patient with a disseminated resistant *Acinetobacter baumannii* infection. *Antimicrob. Agents Chemother*. **61**(10), e00954. Erratum in: *Antimicrob. Agents Chemother*. **62**(12) (2017). 10.1128/AAC.00954-17 PMC5610518 28807909

[38] Gordillo Altamirano FL (2022). Phage-antibiotic combination is a superior treatment against *Acinetobacter baumannii* in a preclinical study. EBioMedicine.

[39] Duyvejonck H (2021). Evaluation of the stability of bacteriophages in different solutions suitable for the production of magistral preparations in Belgium. Viruses.

[40] Lee SJ, Si J, Yun HS, Ko GP (2015). Effect of temperature and relative humidity on the survival of foodborne viruses during food storage. Appl. Environ. Microbiol..

[41] Atamer Z, Hinrichs J (2010). Thermal inactivation of the heat-resistant *Lactococcus lactis* bacteriophage P680 in modern cheese processing. Int. Dairy J..

[42] Müller-Merbach M, Rauscher T, Hinrichs J (2005). Inactivation of bacteriophages by thermal and high-pressure treatment. Int. Dairy J..

[43] Karaböce B (2022). Inactivation of viruses on surfaces by infrared techniques. Int. J. Therm. Sci..

[44] Ochirbat E (2023). Heteroaggregation of virions and microplastics reduces the number of active bacteriophages in aqueous environments. J. Environ. Qual..

[45] Jiang L (2020). Isolation and characterization of a novel myophage Abp9 against pandrug resistant *Acinetobacater baumannii*. Front. Microbiol..

[46] Soontarach R (2022). Isolation and characterisation of bacteriophage selective for key *Acinetobacter baumannii* capsule chemotypes. Pharmaceuticals (Basel).

[47] Selcuk E, Dokuz S, Ozbek T (2024). Evaluating the stability of lytic and lysogenic bacteriophages in various protectants. J. Pharm. Sci..

[48] O'Brien EP, Brooks BR, Thirumalai D (2012). Effects of pH on proteins: Predictions for ensemble and single-molecule pulling experiments. J. Am. Chem. Soc..

[49] Merabishvili M (2017). Stability of bacteriophages in burn wound care products. PLoS ONE.

[50] Xu J (2020). Isolation and characterization of AbTJ, an Acinetobacter baumannii phage, and functional identification of its receptor-binding modules. Viruses.

[51] Feizi S (2021). Colloidal silver combating pathogenic *Pseudomonas aeruginosa* and MRSA in chronic rhinosinusitis. Colloids Surf. B Biointerfaces.

[52] Feizi S (2022). Silver nanoparticles as a bioadjuvant of antibiotics against biofilm-mediated infections with methicillin-resistant *Staphylococcus aureus* and *Pseudomonas aeruginosa* in chronic rhinosinusitis patients. Pathology.

[53] Feizi S (2023). Colloidal silver against macrophage infections and biofilms of atypical mycobacteria. Biometals.

[54] Joshi AS, Singh P, Mijakovic I (2020). Interactions of gold and silver nanoparticles with bacterial biofilms: Molecular interactions behind inhibition and resistance. Int. J. Mol. Sci..

[55] Sharma P, Goyal D, Chudasama B (2022). Antibacterial activity of colloidal copper nanoparticles against Gram-negative (*Escherichia coli* and *Proteus vulgaris*) bacteria. Lett. Appl. Microbiol..

[56] Różańska A, Chmielarczyk A, Romaniszyn D, Majka G, Bulanda M (2018). Antimicrobial effect of copper alloys on Acinetobacter species isolated from infections and hospital environment. Antimicrob. Resist. Infect. Control.

[57] Abdelsattar AS (2022). Utilization of *Ocimum basilicum* extracts for zinc oxide nanoparticles synthesis and their antibacterial activity after a novel combination with phage. Mater. Lett..

[58] Hussain S (2018). Antibiotic-loaded nanoparticles targeted to the site of infection enhance antibacterial efficacy. Nat. Biomed. Eng..

[59] Richter Ł (2021). Broad-spectrum nanoparticles against bacteriophage infections. Nanoscale.

[60] Hao K (2021). Antibacterial activity and mechanism of *Litsea cubeba* L. essential oil against *Acinetobacter baumannii*. Nat. Prod. Commun..

[61] Park DW, Lee JH, Park JH (2021). Thymol and eugenol in essential oils enhance phage endolysin LysECP26-mediated cell wall disruption of *Escherichia coli* O157:H7. Korean J. Food Sci. Technol..

[62] Yue C (2023). Stability of bacteriophages in organic solvents for formulations. Int. J. Pharm..

[63] Johnson G, Wolfe AJ, Putonti C (2019). 2. Characterization of the φctx-like Pseudomonas aeruginosa phage dobby isolated from the kidney stone microbiota. Access Microbiol..

[64] Malki K (2016). Seven bacteriophages isolated from the female urinary microbiota. Genome Announc..

[65] Miller-Ensminger T (2018). Bacteriophages of the urinary microbiome. J. Bacteriol..

[66] Cieślik M (2022). Two newly isolated *Enterobacter*-specific bacteriophages: Biological properties and stability studies. Viruses.

[67] Blazanin M, Lam WT, Vasen E, Chan BK, Turner PE (2022). Decay and damage of therapeutic phage OMKO1 by environmental stressors. PLoS ONE.

[68] Vidakovic L (2023). Biofilm formation on human immune cells is a multicellular predation strategy of *Vibrio cholerae*. Cell.

[69] Betchen M (2022). Evaluating the effectiveness of hospital antiseptics on multidrug-resistant *Acinetobacter baumannii*: Understanding the relationship between microbicide and antibiotic resistance. Antibiotics.

[70] Nawaz R (2023). Development of computationally-guided workflow for designing therapeutic phage cocktail: Targeting multidrug-resistant (MDR) bacteria. Res. Sq..

[71] Cairns BJ, Timms AR, Jansen VA, Connerton IF, Payne RJ (2009). Quantitative models of in vitro bacteriophage-host dynamics and their application to phage therapy. PLoS Pathog..

[72] Merabishvili M (2009). Quality-controlled small-scale production of a well-defined bacteriophage cocktail for use in human clinical trials. PLoS ONE.

[73] Sanchez BC (2022). Development of phage cocktails to treat *E. coli* catheter-associated urinary tract infection and associated biofilms. Front. Microbiol..

[74] Tapia-Rodriguez MR, Cantu-Soto EU, Vazquez-Armenta FJ, Bernal-Mercado AT, Ayala-Zavala JF (2023). Inhibition of *Acinetobacter baumannii* biofilm formation by terpenes from Oregano (*Lippia graveolens*) essential oil. Antibiotics (Basel).

[75] Trafton, A. Using AI, Scientists Find a Drug that Could Combat Drug-Resistant Infections the Machine-Learning Algorithm Identified a Compound that Kills *Acinetobacter baumannii*, a Bacterium that Lurks in Many Hospital Settings. https://news.mit.edu/2023/using-ai-scientists-combat-drug-resistant-infections-0525 (2023).

[76] Zampaloni C (2024). A novel antibiotic class targeting the lipopolysaccharide transporter. Nature.

[77] Pahil KS (2024). A new antibiotic traps lipopolysaccharide in its intermembrane transporter. Nature.

[78] Oleksy-Wawrzyniak M (2021). The in vitro ability of *Klebsiella pneumoniae* to form biofilm and the potential of various compounds to eradicate it from urinary catheters. Pathogens.

[79] Gratia A (1936). Des relations numeriques entre bacteries lysogenes et particles de bacteriophage. Ann. Inst. Pasteur..

[80] Adams MH, Adams MH (1959). Enumeration of bacteriophage particles. The Bacteriophages.

[81] Grygorcewicz B (2021). Environmental phage-based cocktail and antibiotic combination effects on *Acinetobacter baumannii* biofilm in a human urine model. Microb. Drug Resist..

[82] Stepanović S (2007). Quantification of biofilm in microtiter plates: Overview of testing conditions and practical recommendations for assessment of biofilm production by *Staphylococci*. APMIS.

[83] Shahed-Al-Mahmud M (2021). Phage φAB6-borne depolymerase combats *Acinetobacter baumannii* biofilm formation and infection. Antibiotics (Basel).

