# Supplementary material for: Stability study in selected conditions and biofilm-reducing activity of phages active against drug-resistant Acinetobacter baumannii
Source: Sci Rep. 2024 Feb 21;14:4285. doi: 10.1038/s41598-024-54469-z (PMC10881977; doi:10.1038/s41598-024-54469-z)
Supplement: Supplementary file 1 — Supplementary Figures. [file 41598_2024_54469_MOESM1_ESM.docx]

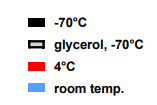
**
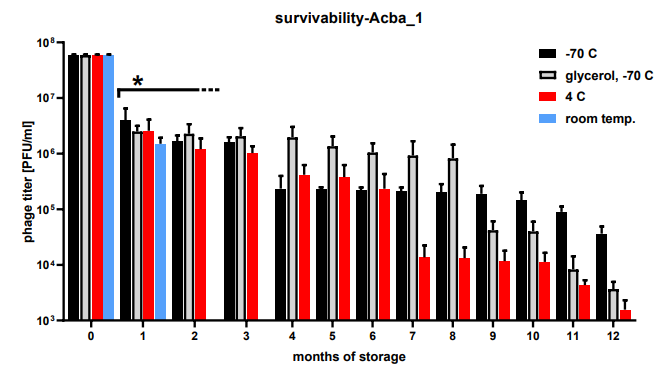
**


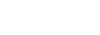

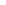


**
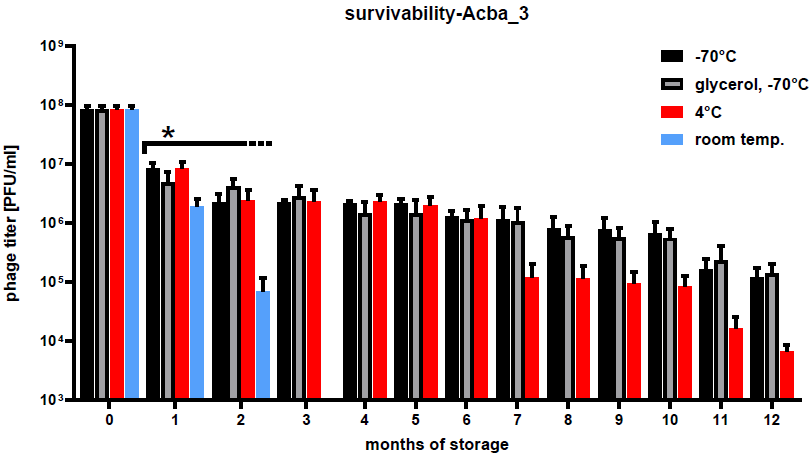
**


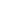

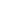

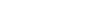

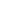


**
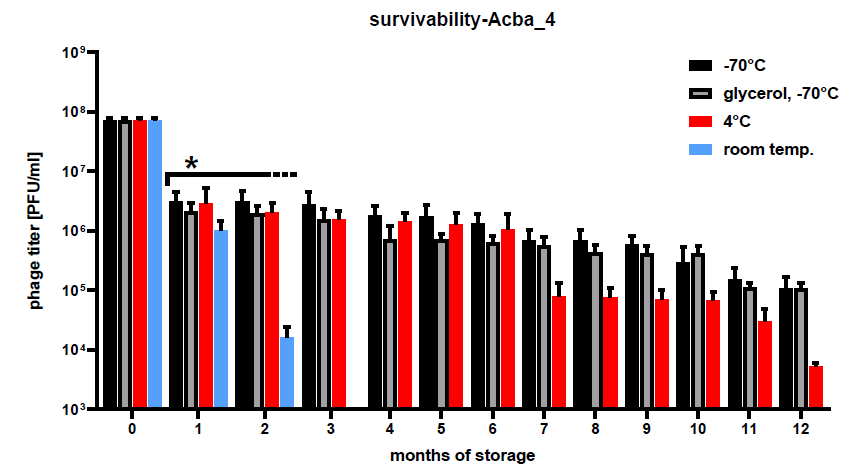
**


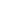

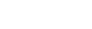

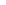


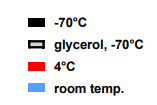
**
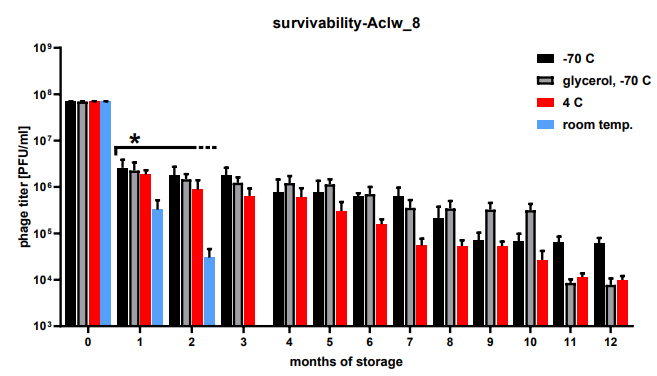
**


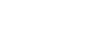

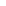


**
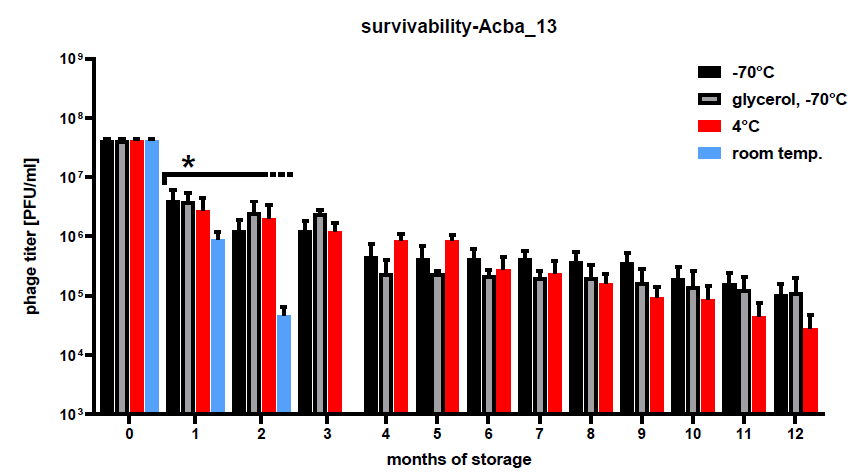
**


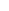

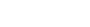

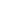

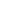


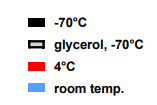
**
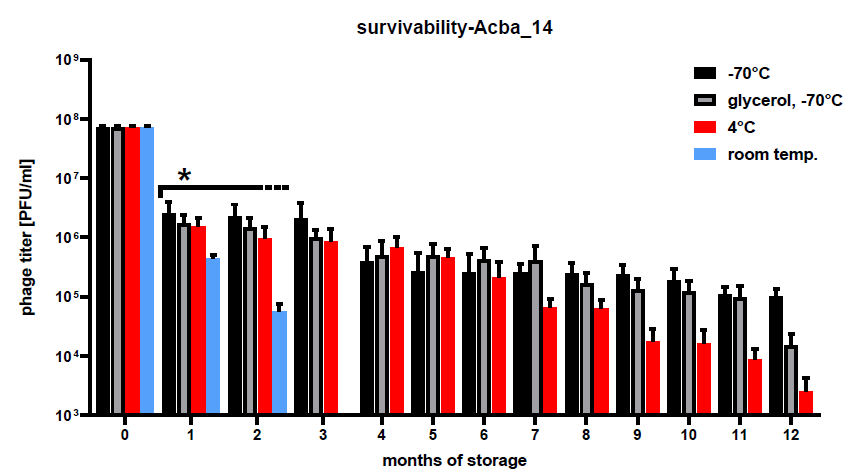
**


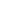

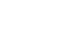

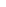


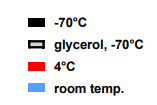
**
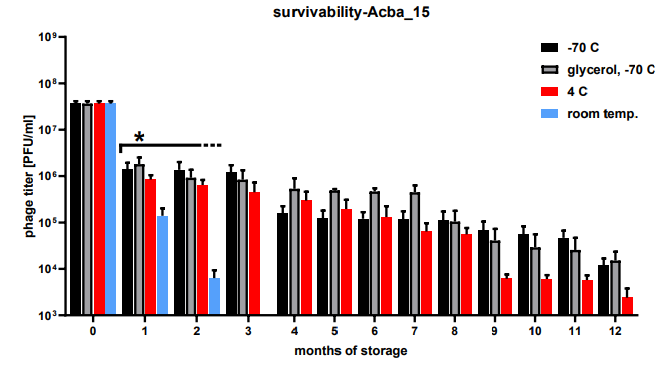
**


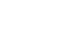

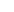


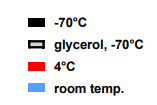
**
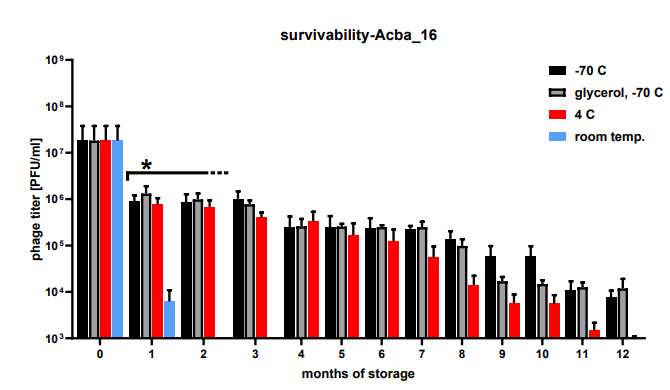
**


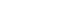

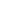


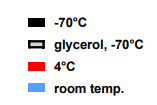
**
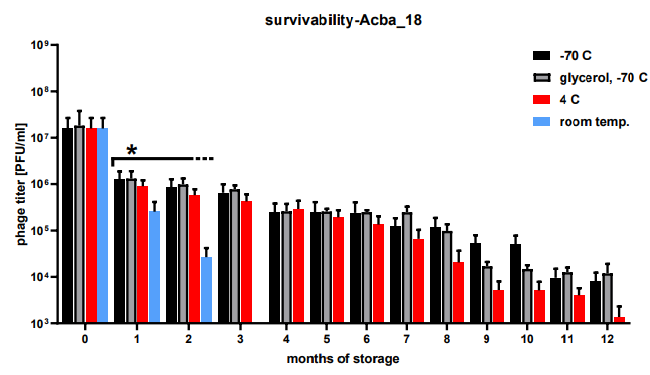
**


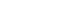

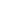

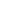


**Figure S1** Stability of phages in different temperature conditions during a 12-month incubation period. Error bars represent the standard deviation (±SD) of the mean phage titers. * p < 0.05.


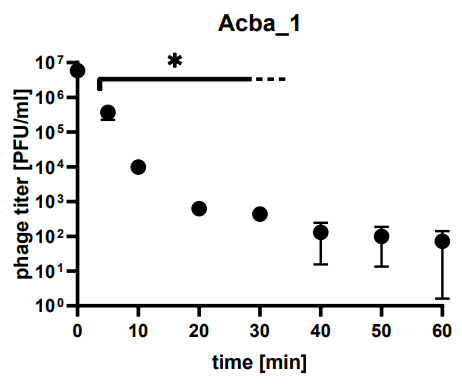


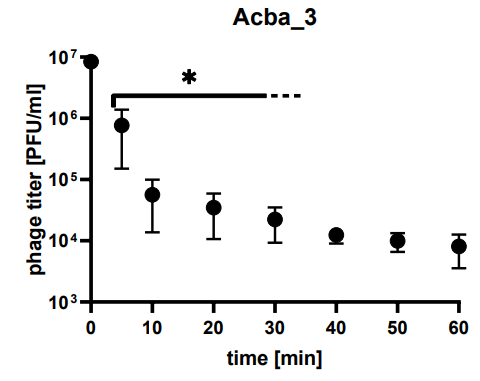


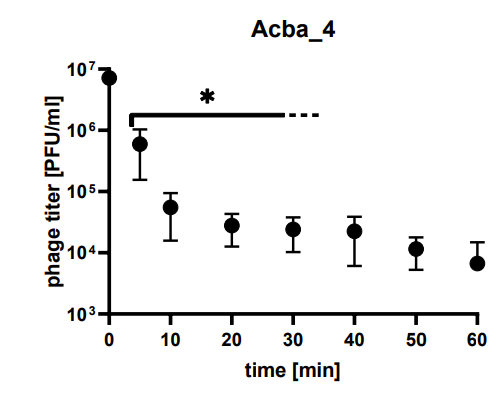


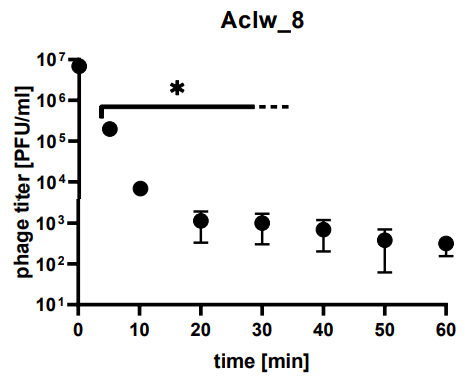


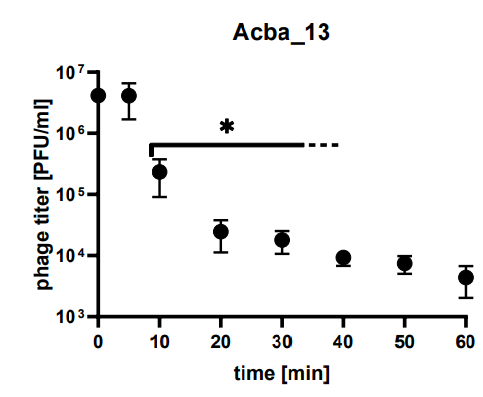


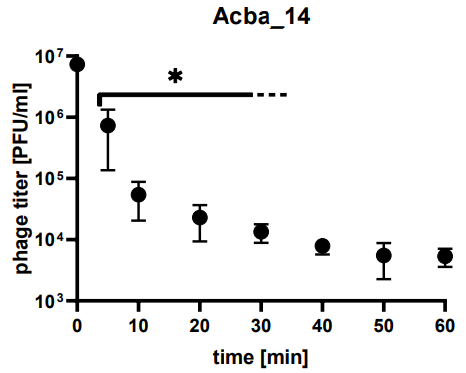


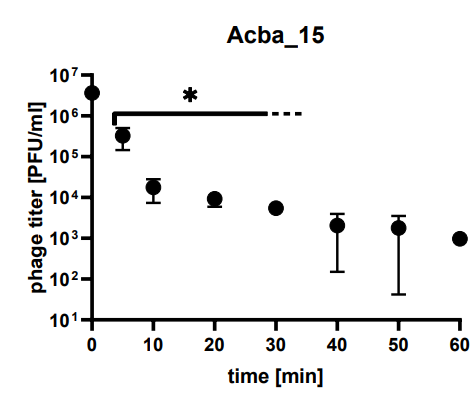


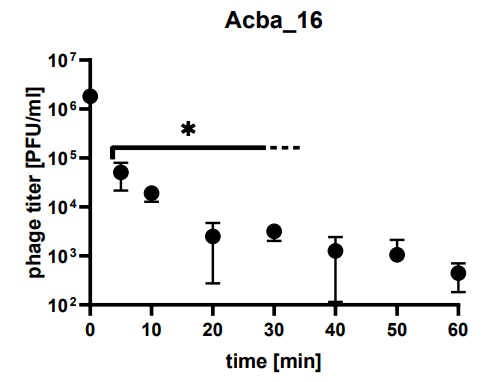


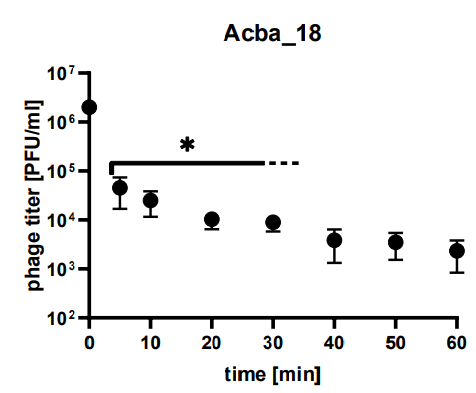


**Figure S2** Stability of phages after one hour of incubation at 60°C. Error bars represent the standard deviation (±SD) of the mean phage titers. * p < 0.05.


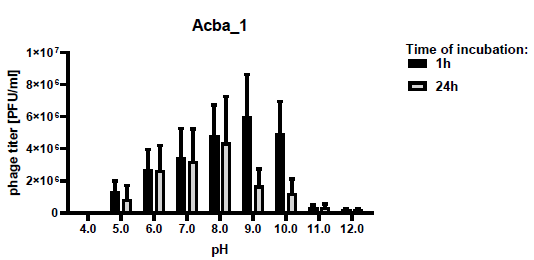


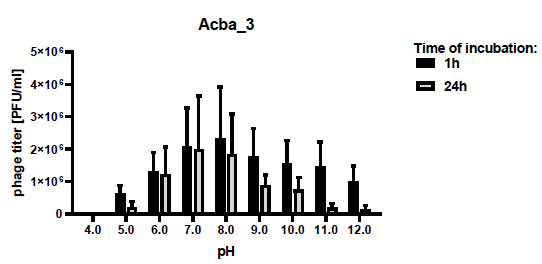


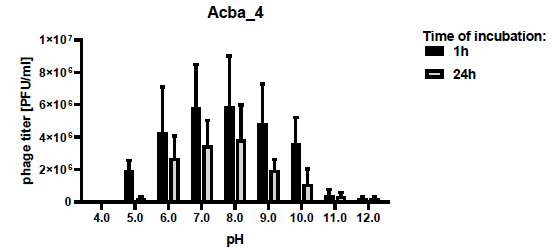


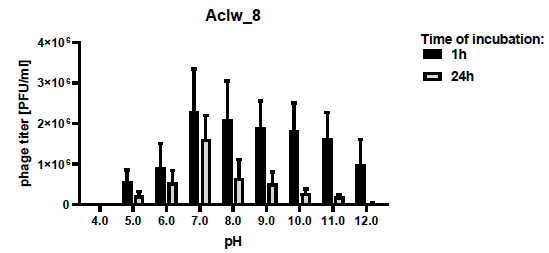


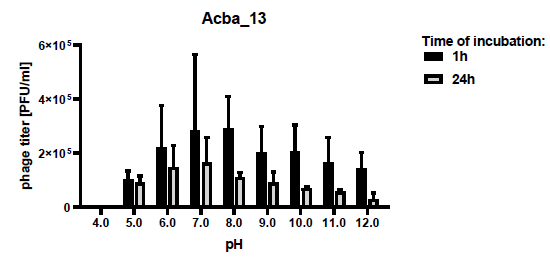


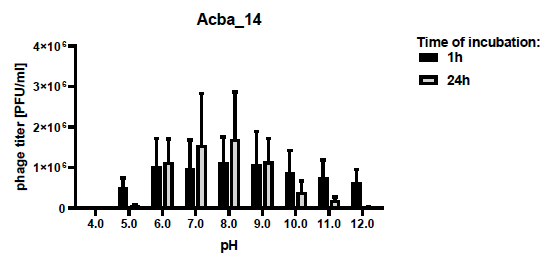


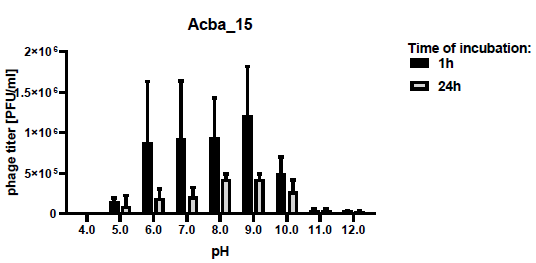


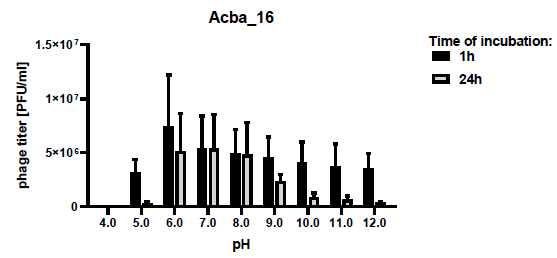


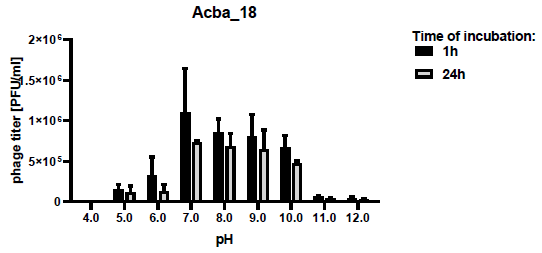


**Figure S3** Stability of phages in various pH conditions during 1h and 24h of incubation. Error bars represent the standard deviation (±SD) of the mean phage titers.


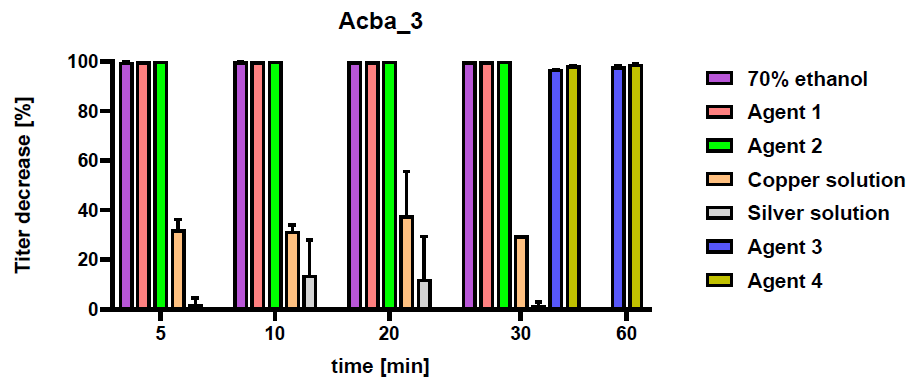


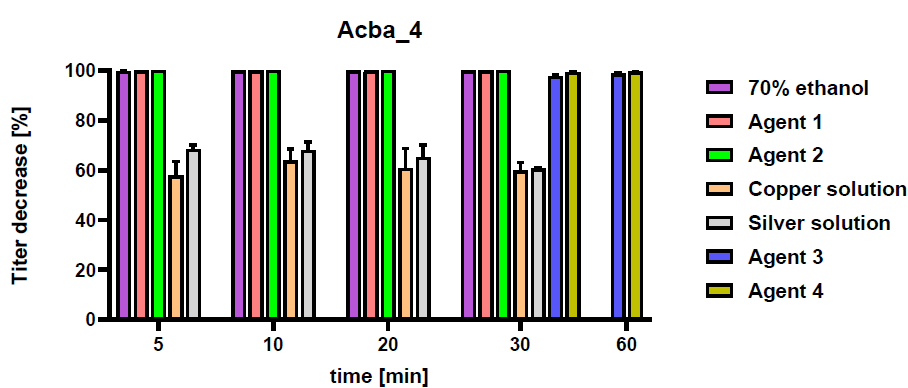


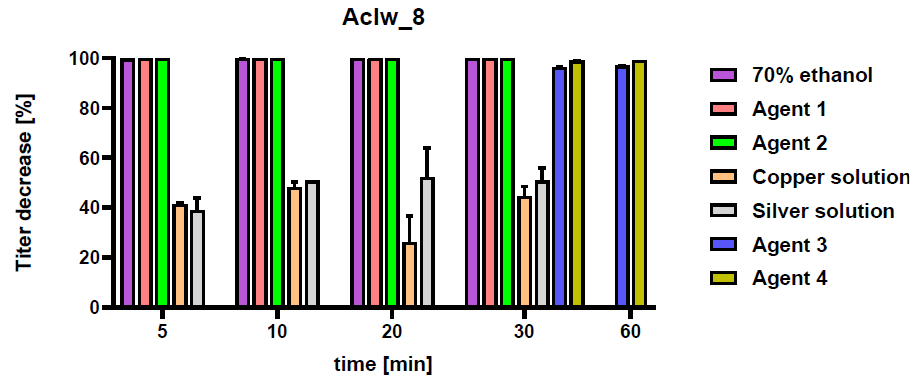


**Figure S4** Percentage decrease in phage titer after incubation in the presence of commonly used disinfectants and metal ions. Error bars represent the standard deviation (±SD) of the mean phage titers.


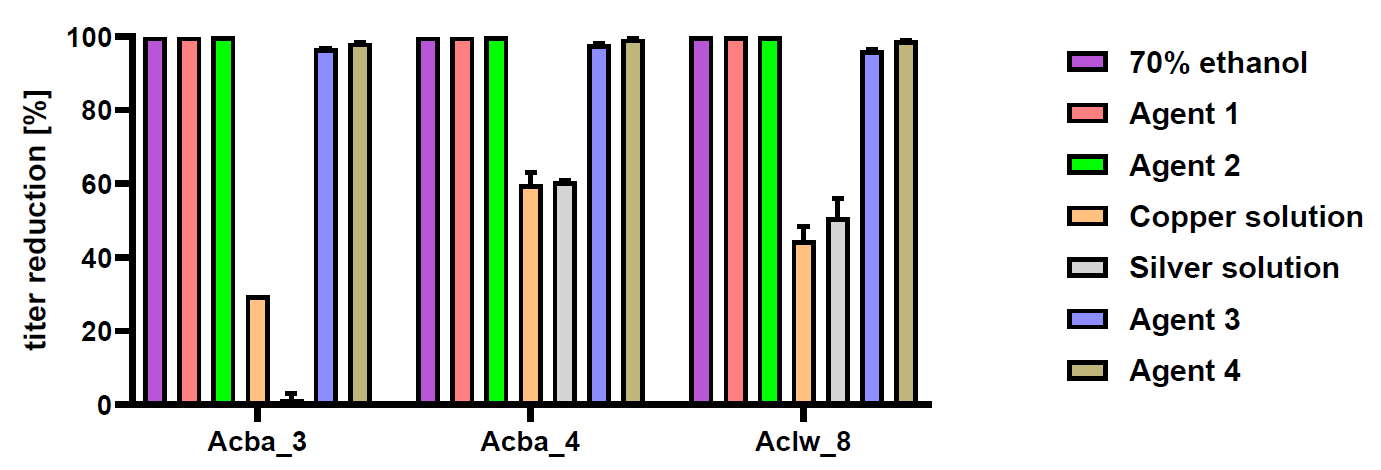

**Figure S5** Comparison of percentage decrease in phage titer after a 30-minute incubation period in the presence of commonly used disinfectants and metal ions. Error bars represent the standard deviation (±SD) of the mean phage titers.

**
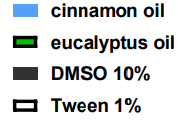
**
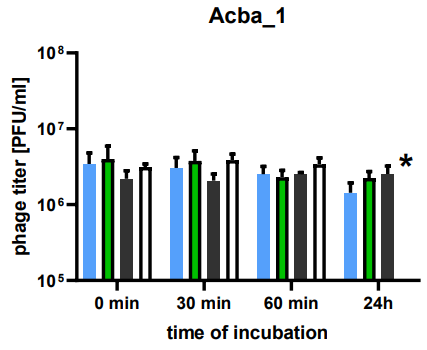


**
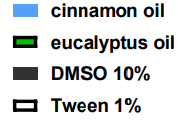
**
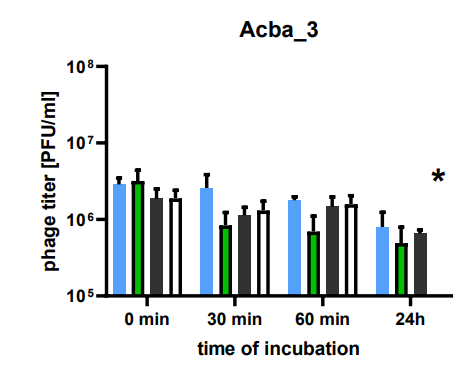


**
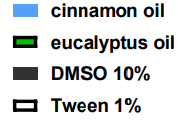
**
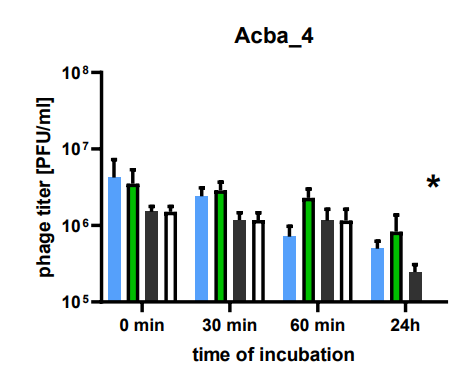


**
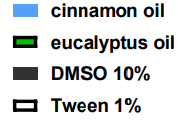
**
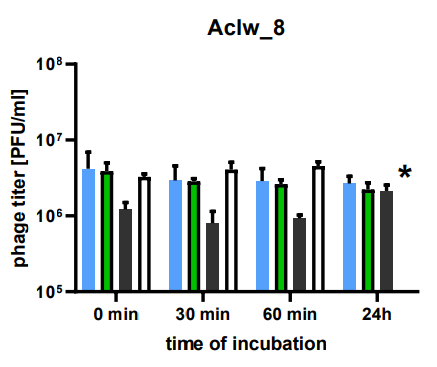


**
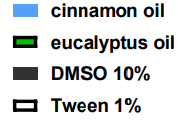
**
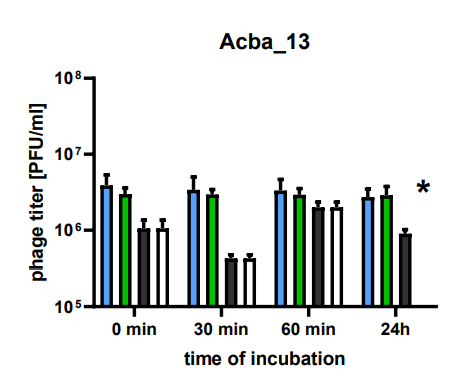


**
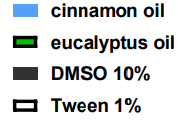
**
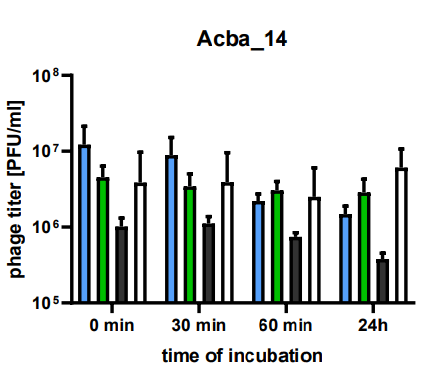


**
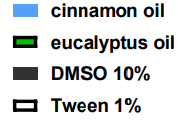
**
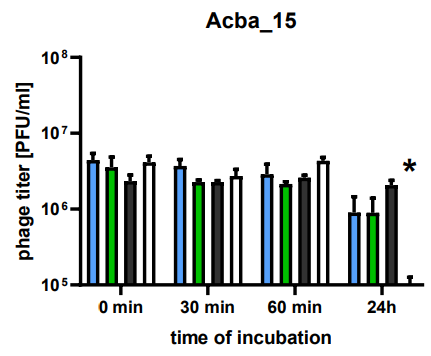


**
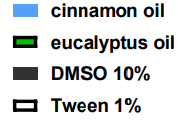
**
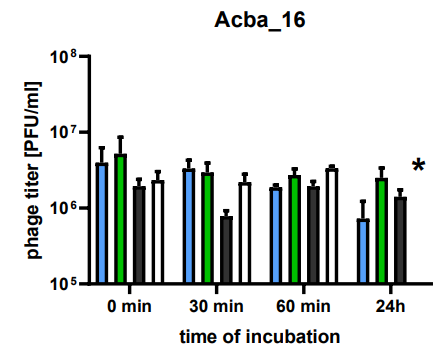


**
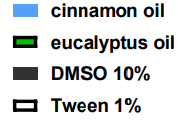
**
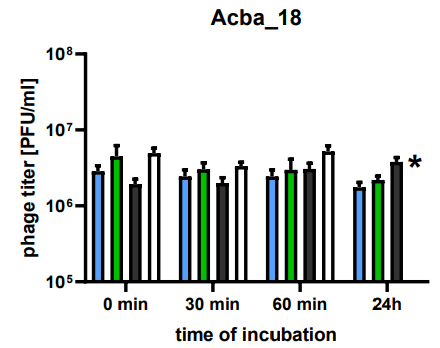


**Figure S6** Stability of selected phages in cinnamon and eucalyptus essential oil, 10% DMSO and 1% Tween-20. Error bars represent the standard deviation (±SD) of the mean phage titers. * p < 0.05.


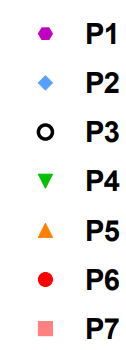

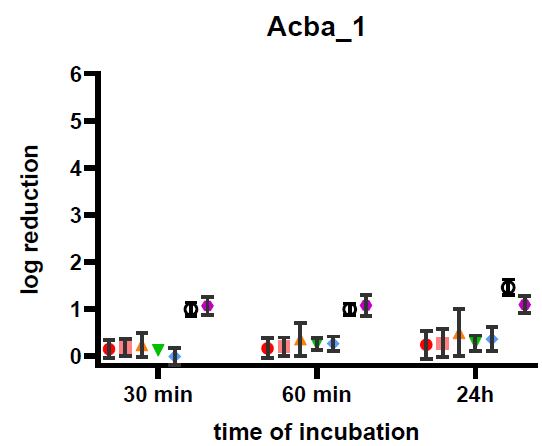


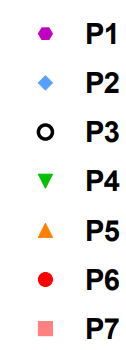

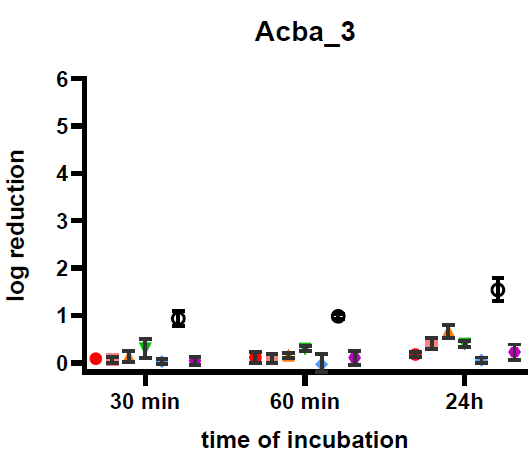


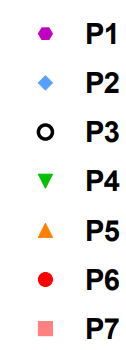

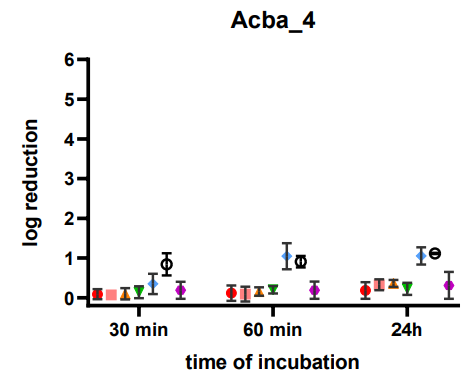


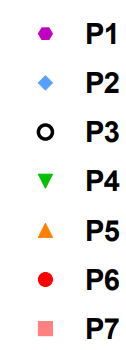

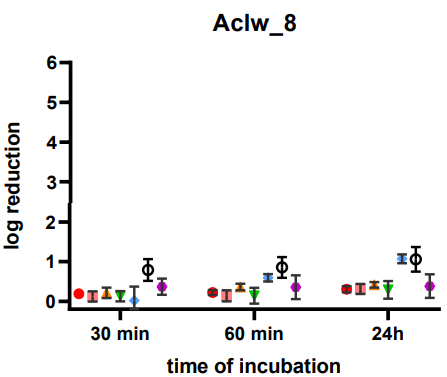


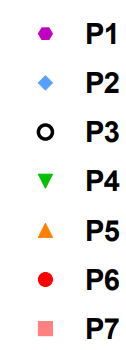

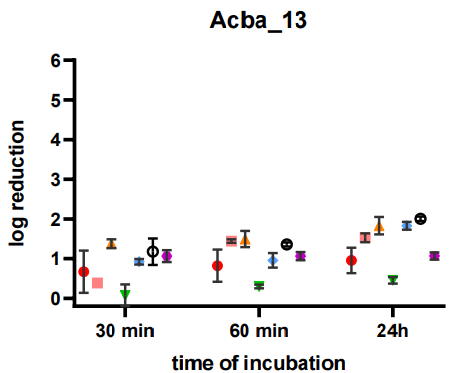


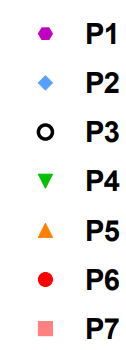

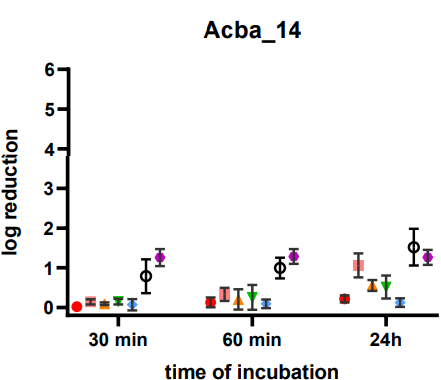


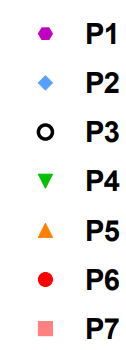

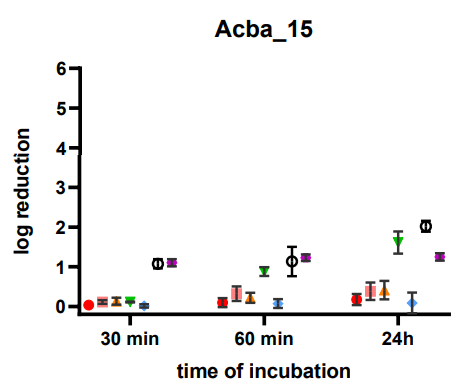


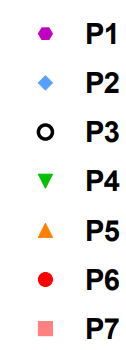

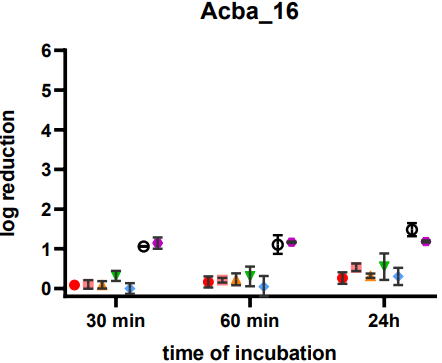


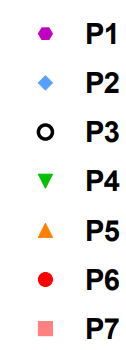

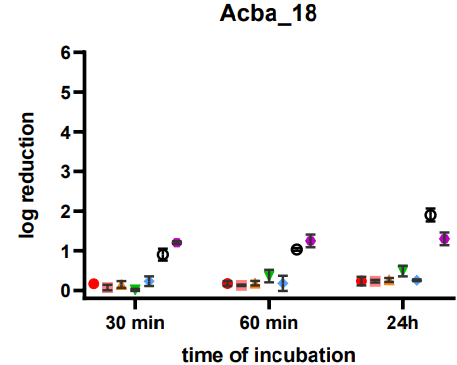


**Figure S7** Stability of selected phages in urine during 30 min, 60 min and 24h of incubation. Points corresponding to 0 min of incubation were used as a control. Error bars represent the standard deviation (±SD) of the mean phage titers.
